# Detecting homologous recombination deficiency for breast cancer through integrative analysis of genomic data

**DOI:** 10.1002/1878-0261.70041

**Published:** 2025-04-22

**Authors:** Rong Zhu, Katherine Eason, Suet‐Feung Chin, Paul A. W. Edwards, Raquel Manzano Garcia, Richard Moulange, Jia Wern Pan, Soo Hwang Teo, Sach Mukherjee, Maurizio Callari, Carlos Caldas, Stephen‐John Sammut, Oscar M. Rueda

**Affiliations:** ^1^ School of Mathematics and Statistics Beijing Institute of Technology Beijing China; ^2^ MRC Biostatistics Unit University of Cambridge UK; ^3^ Cancer Research UK Cambridge Institute, University of Cambridge UK; ^4^ Department of Pathology University of Cambridge UK; ^5^ Cancer Research Malaysia Subang Jaya Malaysia; ^6^ Deutsches Zentrum für Neurodegenerative Erkrankungen (DZNE) Bonn Germany; ^7^ University of Bonn Bonn Germany; ^8^ Fondazione Michelangelo Milano Italy; ^9^ School of Clinical Medicine University of Cambridge UK; ^10^ Breast Cancer Now Toby Robins Research Centre The Institute of Cancer Research London UK; ^11^ The Royal Marsden Hospital NHS Foundation Trust London UK

**Keywords:** breast cancer, cancer genomics, genomic data integration, homologous recombination deficiency, semi‐supervised learning, tumour biomarkers

## Abstract

Homologous recombination deficiency (HRD) leads to genomic instability, and patients with HRD can benefit from HRD‐targeting therapies. Previous studies have primarily focused on identifying HRD biomarkers using data from a single technology. Here we integrated features from different genomic data types, including total copy number (CN), allele‐specific copy number (ASCN) and single nucleotide variants (SNV). Using a semi‐supervised method, we developed HRD classifiers from 1404 breast tumours across two datasets based on their *BRCA1/2* status, demonstrating improved HRD identification when aggregating different data types. Notably, HRD‐positive tumours in ER‐negative disease showed improved survival post‐adjuvant chemotherapy, while HRD status strongly correlated with neoadjuvant treatment response. Furthermore, our analysis of cell lines highlighted a sensitivity to PARP inhibitors, particularly rucaparib, among predicted HRD‐positive lines. Exploring somatic mutations outside *BRCA1/2*, we confirmed variants in several genes associated with HRD. Our method for HRD classification can adapt to different data types or resolutions and can be used in various scenarios to help refine patient selection for HRD‐targeting therapies that might lead to better clinical outcomes.

## Graphical abstract

This study develops a semi‐supervised classifier integrating multi‐genomic data (1404 training/5893 validation samples) to improve homologous recombination deficiency (HRD) detection in breast cancer. Our method demonstrates prognostic value and predicts chemotherapy/PARP inhibitor sensitivity in HRD+ tumours. Its adaptable framework supports diverse data types or resolutions, enabling precise patient selection for HRD‐targeted therapies.

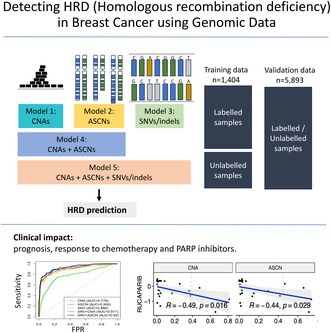

**Abbreviations**
5‐FU: 5‐fluorouracil
ASCN: allele‐specific copy number
AUC: area under the curve
AUCPR: area under the precision‐recall curve
BER: base excision repair
CCLE: cancer cell line encyclopaedia
CHORD: classifier of homologous recombination deficiency
CN: copy number
CNA: copy number aberrations
COSMIC: catalogue of somatic mutations in cancer
DCC: deleted in colorectal carcinoma
DDR: DNA damage response and repair
DNV: double nucleotide variants
DPD: dihydropyrimidine dehydrogenase
EPHA3: ephrin type‐A receptor 3
ER: oestrogen receptor
FDR: false discovery rate
FN: false negatives
FP: false positives
GEL: Genomics England
Her2: human epidermal growth factor receptor 2
HRD: homologous recombination deficiency
HRR: homologous recombination repair
ICGC: international cancer genome consortium
ID: insertions and deletions
ID6: insertions and deletions signature 6
IntClust: integrative clustering
LN: lymph node
LOH: loss of heterozygosity
LOOCV: leave‐one‐out cross‐validation
LST: large‐scale state transitions
MACN: mean altered copy number
MCC: Matthews correlation coefficient
mCN: minor copy number
METABRIC: molecular taxonomy of breast cancer international consortium
MyBrCa: The Malaysian breast cancer study
NHEJ: non‐homologous end joining
Pam50: prediction analysis of microarray 50
PARP: poly ADP ribose polymerase
PCA: principal component analysis
pCR: pathological complete response
PR: progesterone receptor
PRISM: profiling relative inhibition simultaneously in mixtures
RCB: residual cancer burden
RFS: relapse‐free survival
ROC: receiver operating characteristic
SBS: single base substitution
SCAN‐B: Sweden cancerome analysis network – breast
SNV: single nucleotide variants
ssc‐HRD: semi‐supervised classifier of HRD
sWGS: Shallow whole genome sequencing
TAI: telomeric allelic imbalance
TCGA: the cancer genome atlas
TDP: tandem duplication
TN: triple‐negative
TNBC: triple‐negative breast cancer
WES: whole exome sequencing
WGS: whole genome sequencing

## 1 Introduction

DNA double‐strand breaks contribute to the initiation of tumorigenesis and are normally repaired by homologous recombination repair (HRR). Deficiency of this pathway (homologous recombination deficiency, HRD) results in an increased reliance on alternative DNA repair pathways, such as non‐homologous end joining (NHEJ), which are more error‐prone [1]. As a result, HRD leads to the accumulation of somatic mutations, genomic rearrangements, and chromosomal instability [2, 3, 4]. Pathogenic mutations within key genes involved in HRR, including *BRCA1* and *BRCA2*, may result in HRD, and germline mutations in these genes are associated with a significantly increased risk of breast and ovarian cancer [5, 6, 7]. Additionally, promoter DNA hypermethylation of *BRCA1* has been shown to result in HRD [8, 9, 10, 11]. Overall, the biological process of homologous recombination is complex, and HRD can be caused by many different factors that converge on the same phenotype [8].

Identification of HRD status allows the stratification of patients for targeted therapy with poly (ADP ribose) polymerase (PARP) inhibitors, which exploit the dependency of HRD cancer cells on alternative DNA repair pathways, resulting in synthetic lethality [12, 13]. In addition, tumour cells with HRD are often more susceptible to other DNA‐damaging agents, such as platinum‐based chemotherapies [14, 15, 16].

In recent years, much effort has been dedicated to identifying reliable biomarkers to detect HRD in tumours accurately. In the seminal study that defined mutational signatures across the spectrum of human cancer types, single base substitution signature 3 (SBS3) and small insertions and deletions (indels) signature 6 (ID6) were identified as predictors of defective homologous recombination‐based repair [17, 18, 19]. HRDetect [20] integrated features extracted from SNVs, indels and structural variants to develop a logistic regression model to predict HRD, reaching 98.7% sensitivity. Currently, HRDetect is the most sensitive sequencing‐based method for detecting HRD. A similar method, CHORD (Classifier of HOmologous Recombination Deficiency) [21], can discriminate between *BRCA1* and *BRCA2*‐related HRD. Although highly accurate, both methods require whole genome sequencing (WGS) data, which can be expensive and challenging in a clinical setting. Given these limitations, methods that use copy number aberrations (CNA) to characterise HRD‐related profiles have been developed. However, these methods predate sequencing‐based predictors. A shrunken centroids classifier [22] was developed based on 371 genomic regions to detect what was termed ‘BRCAness’ [8]. This classifier showed robustness in estimating HRD on different CNA estimation platforms [23]. Additionally, incorporating information from allele‐specific copy number (ASCN) further improved HRD detection. The term ‘genomic scars’ [24] was defined based on three quantitative indicators: loss of heterozygosity (LOH) [25], telomeric allelic imbalance (TAI) [26], and large‐scale state transitions (LST) [27]. The integration of these three indicators using the average of the three individual scores predicted deficiencies in DNA damage repair within different subtypes of breast cancer [28]. A subsequent study identified a cut‐off value for the sum of LOH, TAI and LST scores [16]. This method, named scarHRD, demonstrated a strong correlation between HRD scores derived from SNP array‐based and next‐generation sequencing‐based approaches [29]. More recently, copy number signatures [30], such as CX3, have been proposed as another biomarker for HRD. Moving from DNA, other data types have also been successful in estimating HRD tumours, such as gene expression for triple‐negative (TN) breast tumours [31], a 228‐gene transcriptomic signature [32], or *RAD51* foci [33].

All these methods characterise different features that may require various assays with variable associated costs, such as structural variant features from WGS, mutational signatures from whole exome sequencing (WES), and copy number features from SNP arrays, WES, or shallow whole genome sequencing (sWGS). Here, we describe the development of a multi‐modal classifier using a semi‐supervised random forest‐based self‐training method that combines DNA data from different platforms. We labelled *BRCA1/2*‐defective tumours as HRD+ as this is the most common mechanism that produces the phenotype. However, as mentioned, many other genes in the pathway and other aberrations different from single nucleotide variations can cause HRD. For this reason, features that capture the genomic profile of these tumours might be more successful in identifying the phenotype than the status of specific genes. However, in order to compare different models and methods in the most unbiased way, we used *BRCA1/2* status as the gold standard, as this is not a feature used in any of the models compared in this work.

Instead of developing a single model, we consider increasingly complex models that integrate features from different sources, such as SNP arrays, WES, or WGS. Thus, the method adapts to the type of data available. Using several breast cancer cohorts, we find that our classifier has a similar performance to other alternatives that detect *BRCA1/2*‐defective tumours. As expected, models that incorporate more information produce more accurate HRD identification. We also quantify the increase in performance that comes with more comprehensive genomic characterisation, providing a potentially valuable methodological framework for exploring decisions in clinical contexts, especially considering the resource constraints. Integration of data from different sources and profiling from various platforms allows us to find associations between features from different data types and reveal hidden connections between features to improve our understanding of the HRD mechanism.

## 2 Methods

### 2.1 Training and validation datasets

To account for variability in genomic assays, pre‐processing pipelines, bioinformatic methods, and cohort composition, two distinct datasets were used to train our model: TCGA (The Cancer Genome Atlas Breast Invasive Carcinoma [34], *n* = 1003) and ICGC (The International Cancer Genome Consortium Breast Cancer [35], *n* = 401).

We validated our classifiers in additional datasets such as GEL (Genomics England Breast Cancer [36], *n* = 2964), SCAN‐B (Sweden Cancerome Analysis Network–Breast [37, 38], *n* = 231), TransNEO (a neoadjuvant study [39], *n* = 164), MyBrCa (The Malaysian Breast Cancer study [40], *n* = 525), METABRIC (Molecular Taxonomy of Breast Cancer International Consortium [41, 42, 43], *n* = 2009), and CCLE (the Cancer Cell Line Encyclopaedia [44, 45], *n* = 853 for normalisation, and *n* = 25 for breast cancer cell lines with drug sensitivity assessed using the PRISM metric).

Table S1 provides a detailed description of the composition of all cohorts, including the data source, reference genome, sequencing technology used for SNV and ASCN, sample size, as well as the number and proportions of samples classified as HRD+, HRD−, and HRD unknown. Full details can be obtained in the corresponding papers describing each cohort.

In the determination of HRD status, we followed the labelling convention employed by HRDetect [20]. A sample was classified as HRD‐positive (HRD+) when it met any of the following criteria:

- Pathogenic germline *BRCA1/2* mutations coupled with the loss of the second allele/loss of heterozygosity (LOH);
- Somatic *BRCA1/2* mutations accompanied by LOH;
- Promoter DNA hypermethylation of *BRCA1* in conjunction with LOH.

Conversely, HRD‐negative (HRD−) cases were identified when there were no detected *BRCA1/2* mutations or when both *BRCA1/2* genes exhibited no LOH. The rest of the samples could not be accurately categorised and were labelled as unknown.

### 2.2 Data normalisation and feature selection

We integrated data from our two training cohorts together for model development. However, within each cohort, each data type was normalised. This was accomplished through log scale transformation according to the following formula $\left(x-\text{mean}\left(x\right)\right)/\mathrm{sd}\left(x\right)$, where $x=\ln\left(\text{feature}+1\right)$ is the log‐transformed feature.

The first model exclusively incorporated features derived from CNA data with no allele‐specific information. These included six distinct CNA scores (cna burden, cna load, mean altered copy number, tandem duplication score, tandem duplication region size and chromothripsis score) obtained from the ‘scoring’ function within the R package ‘sigminer’ [46]. These scores provided a quantitative assessment of the copy number profile and associated events. The cna burden signified the proportion of the genome that had undergone genomic alterations, while the cna load represented the quantity of copy number alterations. The mean altered copy number (MACN) characterised the average value of altered copy number segments. The tandem duplication score (TDP) assessed the presence of tandem duplications, and TDP region size per megabase (Mb) offered insight into the size distribution of tandem duplication regions. And the chromothripsis state score provided a measure of the chromothripsis state within the genome. In addition, we considered six breast cancer‐specific copy number signatures (CX1, 2, 3, 4, 5, and 9) [30]. These features can be computed from affordable assays, such as shallow WGS or aCGH arrays. We used the ‘quantifyCNSignatures’ function in the ‘CINSignatureQuantification’ package for quantification. During this process, we specifically set cancer.subset = ‘BRCA’ to ensure the optimal estimation for breast cancer samples.

The second model included all previous 12 CNA features from the first model and added 9 additional features based on ASCN data. These additional features relied on LOH estimation and could be obtained from SNP arrays, WES or WGS. Regarding the breast cancer‐specific CN signatures in Steele et al. (2022) [47], we first applied the ‘getMatrix’ function in the ‘panConusig’ package (see the detailed code at: https://github.com/UCL‐Research‐Department‐of‐Pathology/panConusig/blob/main/R/setup_CNsigs.R) to obtain a catalogue covering 48 CN signatures, then we relied on the ‘sig_fit’ function in the ‘sigminer’ package to obtain the exposures of CN signatures. Based on the breast cancer‐specific CN signature information from Figure 2 [47], we only considered these breast cancer‐specific copy number signatures (CN1, 2, 6, 7, 8, 9, 11 and 17). We also included the scarHRD score described previously.

The third model used SNV and indel data, taken from WES or WGS. These features included 12 breast cancer‐specific single base substitution signatures (SBS1, 2, 3, 5, 6, 8, 13, 17b, 18, 20, 26 and 30), along with 18 small insertions and deletions signatures (ID1‐18) sourced from cosmic (Catalogue of somatic mutations in cancer) version 3.3. We utilised the ‘sig_fit’ function in the ‘sigminer’ package to obtain the exposures of these SBS and ID signatures.

The fourth model integrated data from CNA and SNV for cases where LOH estimation was not robust (such as low depth WES or shallow WGS).

Finally, the fifth model integrated all features from ASCN and SNV.

Table S2 offers a brief explanation of each feature, while Fig. S1 and Table S3 provides a list of the five trained models. These were categorised based on the features they incorporate, offering a clear overview of the models developed in this study.

Firstly, to screen out features with statistically significant differences, we performed a Wilcoxon test between HRD‐positive and HRD‐negative samples. Specifically, we calculated the Wilcoxon rank sum test *P*‐value for each feature. If a feature's *P*‐value was >0.05, it was excluded from further analysis. After this step, 32 statistically significant features were retained, which were further categorised by data type into CNA, ASCN, and SNV, with 10, 5 and 17 features retained respectively. We did not adjust the *P*‐values for multiple comparisons, as this step was intended as a preliminary screening to retain candidate features and avoid missing potentially useful ones.

To address potential collinearity issues, we then conducted pairwise comparisons of the retained features within the CNA, ASCN and SNV datasets by calculating Pearson's r correlation coefficients. When the correlation coefficient between a pair of features exceeded 0.8, based on certain biological rationales and research background knowledge, we selectively removed one of the features. For example, among the 10 retained features in the CNA dataset, the correlation between ‘tandem duplication region size’ and ‘cna load’ was as high as 0.908, and its correlation with ‘CX5’ was 0.860. Considering that we had already retained the ‘tandem duplication score’ related to ‘tandem duplication’ and that previous research had indicated a close association between ‘CX5’ and HRD, we decided to remove ‘tandem duplication region size’, resulting in 9 features remaining in the CNA dataset. Similarly, in the ASCN dataset, the pairwise correlations of the 5 retained features were all <0.8, so they were all kept; in the SNV dataset, the correlation between ‘SBS2’ and ‘SBS13’ was 0.807. Given that both were related to the ‘activity of the AID/APOBEC family of cytidine deaminases’ and as documented in the COSMIC dataset (e.g. https://cancer.sanger.ac.uk/signatures/sbs/sbs2/), ‘SBS2’ usually appeared in the same samples as ‘SBS13’ and was closely related. To avoid collinearity, we removed ‘SBS2’, which had a relatively larger Wilcoxon rank sum test *P*‐value, and finally 16 features were retained in the SNV dataset. In summary, after careful screening and consideration, we ultimately retained 30 key features (Fig. S2), with 9, 5 and 16 features in the CNA, ASCN and SNV datasets respectively.

### 2.3 Semi‐supervised classifier for HRD status

To extract information from both the labelled and unlabelled cases, we used a semi‐supervised approach based on a random forest self‐training method implemented in the ‘selfTraining’ function from the R package ‘ssc’ [48]. The classifier was initially trained on a subset of labelled samples, utilising a random forest classifier. Subsequently, it entered an iterative process where it was repeatedly retrained using its own most confident predictions on the unlabelled instances. Self‐training adhered to a wrapper methodology that leverages a base supervised classifier to ascertain the probable class assignments for unlabelled data points.

Obtaining labels for data could often be an expensive and time‐consuming process, particularly in the context of obtaining HRD labels, where determining the germline and somatic *BRCA1/2* mutation status plus LOH was imperative for each sample. Furthermore, HRD‐positive was attributed to various factors, including abnormalities in chromosome structure, particularly deletions of long or short arms on chromosomes [32, 49], which could impede the progression of homologous recombination. Disruptions in DNA methylation patterns might also adversely affect the execution of homologous recombination. The association of HRD with irregularities in cell cycle regulatory pathways was noteworthy, as homologous recombination typically transpires during specific phases of the cell cycle. Beyond the well‐established roles of *BRCA1* and *BRCA2*, there exists a spectrum of other genes that might contribute to HRD, particularly those intricately involved in DNA repair mechanisms and the control of cell cycle progression. In addressing this challenge, semi‐supervised learning emerged as a valuable approach, leveraging training from a limited set of labelled data in conjunction with an extensive pool of unlabelled data. Semi‐supervised classification occupied an intermediate position between fully supervised and unsupervised classification methodologies. By adopting a semi‐supervised strategy, a classifier could be trained on a small subset of labelled data, subsequently utilising the classifier to make predictions on the larger pool of unlabelled data. Given that these predictions surpassed random guessing, the resultant unlabelled data predictions could be integrated as ‘pseudo‐labels’ in successive iterations of the classifier. The concept of self‐labelling techniques [50] was designed to enrich the original labelled dataset by incorporating the most confident predictions for classifying unlabelled data. Among these techniques, the self‐training method stood out as one of the most prominent. The self‐training implementation employed in this study was grounded in Yarowsky's algorithm [48]. To stop the self‐labelling process, the stopping criteria entailed iterating through the process until a predetermined portion of the unlabelled set had been exhausted.

In the context of a binary classification task, the selection of a threshold was a pivotal step in mapping the model's output into distinct categories. The default choice of 0.5 was grounded in its intuitive interpretation—when the output probability surpassed 0.5, it was categorised as positive; otherwise, it was deemed negative. However, in scenarios where the distribution of positive and negative HRD samples was highly imbalanced, adherence to the 0.5 threshold might result in the model allocating insufficient attention to the minority HRD‐positive categories. By fine‐tuning the threshold, a better adaptation to imbalanced data was achievable, striking a balance between precision, recall, F1 score, and other performance metrics to meet specific requirements. To optimise model performance, we employed a Leave‐One‐Out Cross‐Validation (LOOCV) approach for threshold selection. Specifically, we instantiated five models: CNA, ASCN, SNV, SNV + CNA, and SNV + ASCN. Each model incorporates a distinct set of features, comprising 9, 14, 16, 25 and 30 features, respectively. One sample was systematically omitted at a time, and the remaining 1403 samples were incorporated into the models using the already described random forest‐based self‐training method. Subsequently, the model predicted the omitted sample, and this process iterated 1404 times until all samples had been excluded. The predicted probabilities from each iteration were amalgamated. Through the computation of the maximum F1 score, optimal thresholds for the five models were determined, yielding values of 0.382, 0.450, 0.344, 0.296 and 0.374 for these five models, respectively. This was computed using a custom function in R.

After internal validation, we used all tumours in our two cohorts to develop the definite five models. Table S4 presents the predicted HRD probabilities in five models for all training and validation cohorts.

### 2.4 Statistical analysis

#### 2.4.1 Importance scores computation

Importance scores for each feature were obtained using the ‘importance’ function from the ‘randomforest’ package in R [51].

#### 2.4.2 Area Under the Curve (AUC) computation

AUC and AUC precision‐recall (AUCPR) values were calculated with the ‘roc.curve’ and ‘pr.curve’ functions available in the R package ‘PRROC’ [52].

#### 2.4.3 Principal component analysis (PCA)

To investigate whether the genomic features of samples labelled as true HRD− (based on *BRCA1/2* status) but classified as HRD+ by our model and the scarHRD method resemble those of true HRD+ samples, we performed a PCA across all features based on samples from the combined cohorts, excluding those with unknown HRD status. The first two principal components (PC1 and PC2) were calculated with the ‘prcomp’ function available in the R package ‘stats’.

#### 2.4.4 Odds ratios from multivariable logistic models

To evaluate whether the predicted HRD probabilities can be used to predict pathological complete response (pCR), we constructed multivariable logistic models using the TransNEO dataset. The models included ER status, lymph node status, and tumour grade–consistent with the clinical baseline model described in the original paper [39]. Additionally, we incorporated the predicted HRD probabilities from our five models. Using pCR = 0 as the reference group, we obtained the odds ratios for these features across the five multivariable logistic models.

#### 2.4.5 Cox proportional hazards models

To assess the association between our HRD status predictions and adjuvant chemotherapy response, we focused on a subgroup consisting of 375 individuals who underwent chemotherapy in the METABRIC dataset. Using endpoint relapse‐free survival (RFS), we fitted five independent univariable Cox models for Her2+, ER+/Her2− (HR+) and ER−/Her2− (TNBC) tumours separately, using the probabilities of HRD provided by each of our semi‐supervised models. During this process, we initially explored potential confounders by performing univariable Cox analyses on clinical variables including age, lymph node status, tumour stage and grade (see Table S5). As none of these variables (except stage IV) showed a significant association with relapse‐free survival, we did not include them in the final model, aiming to focus on the impact of the HRD probability output by the model itself on survival outcomes.

#### 2.4.6 Fisher's test

Fisher's test for association between genomic events and HRD status: Using a curated list of 781 genes related to cancer and the homologous recombination pathway, we employed Fisher's exact test to assess the association between the presence of a somatic mutation in these genes and the predicted HRD status among the five models in our training cohorts. Copy numbers were classified into three states: loss, neutral, and gain, represented by −1, 0 and 1, corresponding to copy numbers of <2, =2 and >2, respectively. *P*‐values were adjusted for multiple testing using the false discovery rate (FDR) correction method (Benjamini‐Hochberg procedure).

#### 2.4.7 Comparison with gradient boosting and logistic regression

We employed a semi‐supervised random forest‐based self‐training method in our study. To assess the robustness of the semi‐supervised approach, we also considered semi‐supervised gradient boosting and logistic regression‐based self‐training methods. Figure S3 presents the Receiver Operating Characteristic (ROC) curves for LOOCV predicted probabilities from five models, which are based on HRD status within both the combined cohorts and each individual cohort, using the semi‐supervised random forest‐based, gradient boosting, and logistic regression‐based self‐training methods. Upon comparison, we observe that the AUC values for the five random forest models are consistently higher than those for the other methods, except in one instance (CNA). However, it is also noteworthy that the differences among these three methods are relatively small. This is a desirable outcome, as it suggests that the semi‐supervised method remains robust regardless of the chosen classifier.

## 3 Results

**Fig. 1:**
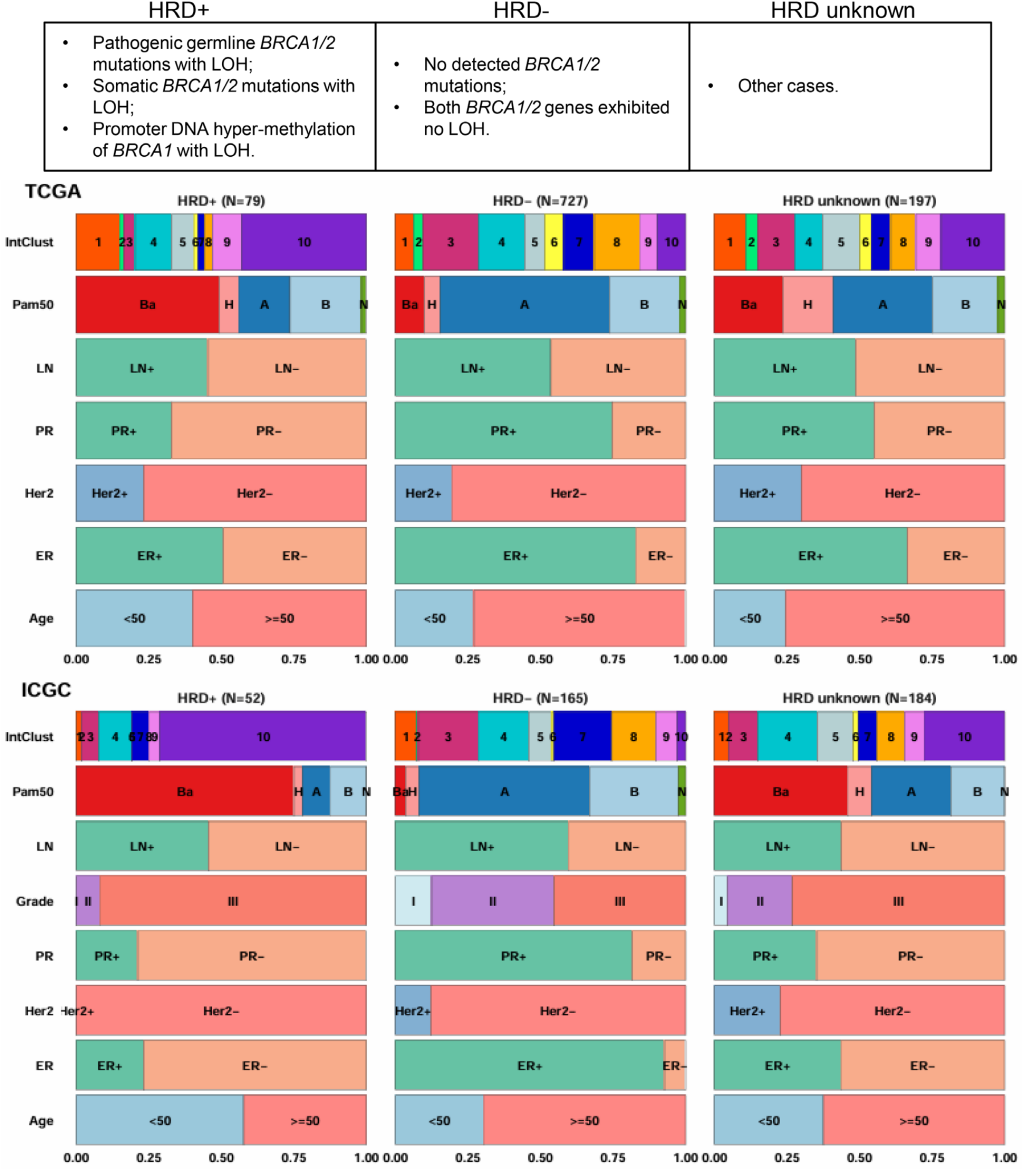
Criteria to identify HRD+ and HRD− tumours and characteristics of tumours stratified on HRD‐positive (HRD+), HRD‐negative (HRD−), and unknown HRD status. Subtypes in the Pam50 (Prediction analysis of microarray 50) classification system are abbreviated as Ba (basal‐like), H (Her2‐enriched), A (luminal A), B (luminal B), and N (normal‐like). ER, oestrogen receptor; Her2, Human epidermal growth factor receptor 2; IntClust, integrative clustering; LN, lymph node; LOH, loss of heterozygosity; PR, progesterone receptor.

Combining data from two different breast cancer cohorts (*n* = 1404 tumours), we labelled HRD status following the same methodology employed by the HRDetect algorithm, which was based on the status of *BRCA1/2* [20]. Figure 1 and Table S1 summarise the criteria for determining HRD+/− samples and their distribution in each cohort.

**Fig. 2:**
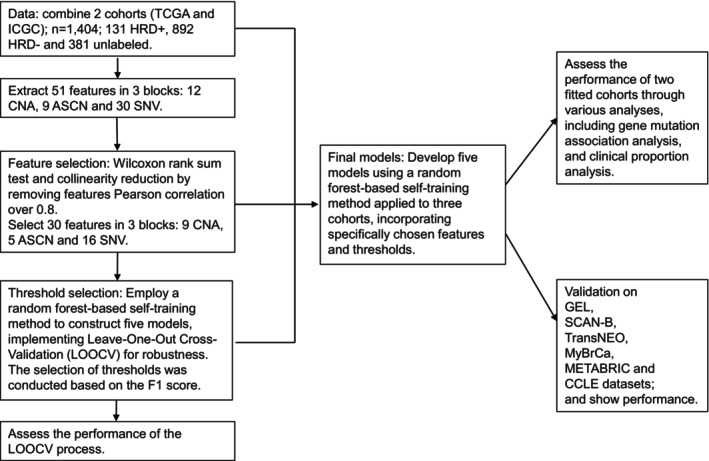
Model construction strategy.

Given that mutations in HRR pathways induce genome instability, resulting in copy number aberrations, loss of heterozygosity and somatic mutations, our aim was to extract HRD‐related information from data obtained from various sources. We adopted a multi‐source data strategy that integrated information from three distinct data blocks: copy number aberrations (CNA), allele‐specific copy number (ASCN) and single nucleotide variants and indels (SNV). Within this framework, we developed five potential models, each progressively incorporating an increasing number of integrated features. These models were constructed by selectively combining features from these three data blocks, resulting in the following categories: CNA, ASCN, SNV, SNV + CNA and SNV + ASCN. Model construction is shown in Fig. 2.

Feature selection for each model is described in the Methods section. The full list of features is presented in Table S2, while Fig. S1 and Table S3 provide a summary of the five trained models. These were categorised based on the features they incorporated, offering a clear overview of the models developed in this study. After testing for statistical significance and removal of correlated features (see Methods section), 30 features across all models were retained.

Since it was not possible to determine the *BRCA1/2* status for many samples due to incomplete information—such as the absence of data on pathogenic germline *BRCA1/2* mutations, somatic *BRCA1/2* mutations, *BRCA1* promoter DNA hypermethylation, or *BRCA1/2* LOH status—we used a semi‐supervised approach with a random forest‐based self‐training method, implemented in the R package ‘ssc’ [50]. This methodology allowed us to incorporate samples of unknown HRD status in the inference process; at the same time, estimating the parameters for each class while evaluating the confidence in predicting the HRD status of the unlabelled samples. The model's output was the probability for a tumour to be HRD+. However, in clinical applications, patients need to be assigned to one of the two categories. To select the optimal threshold to call HRD+ samples, we employed a LOOCV approach (see Methods).

**Fig. 3:**
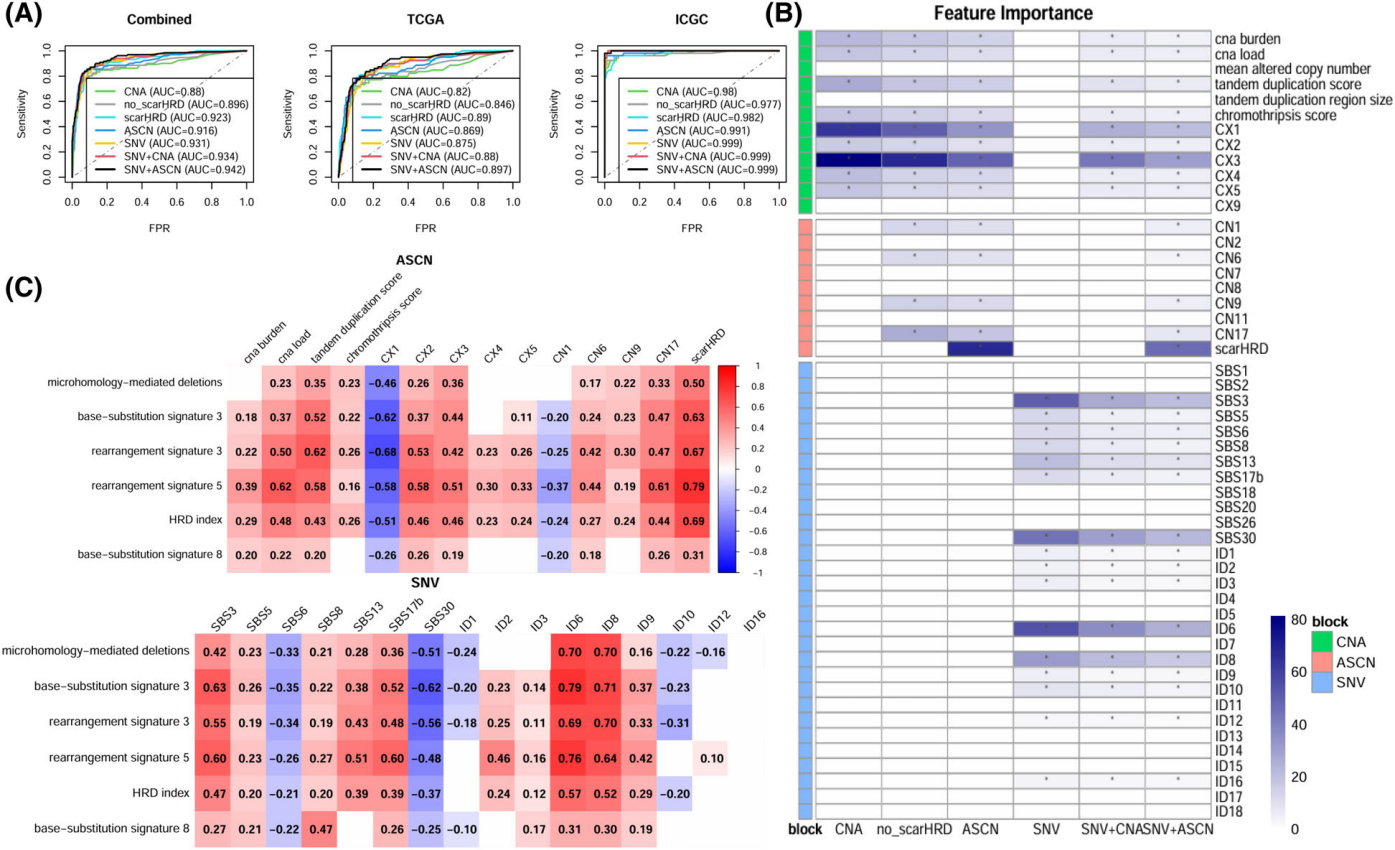
(A) Receiver Operating Characteristic (ROC) curves for Leave‐one‐out cross‐validation (LOOCV) predicted probabilities in seven models based on the HRD status within the combined two cohorts and each cohort. The no_scarHRD model incorporates relative copy number features and ASCN features while excluding the scarHRD score, whereas the scarHRD model consists solely of the scarHRD score. (B) Feature importance of six models in all features from three blocks, where features in cells marked with an asterisk (*) are selected. (C) Pearson's product moment correlation coefficient between six features of HRDetect and selected features of three blocks in the ICGC set. Non‐significant results based on a correlation test are blank.

We assessed the performance of the models comparing their predictions and the *BRCA1/2* status using the AUC and the AUCPR values. Figure 3A and Fig. S4 illustrate the curves for the five models in each cohort. In the combined dataset, we observed a range of AUC values from 0.880 to 0.942 depending on the list of features used. As the models incorporated more complex features, starting with only log_2_‐ratio copy number data and progressively including additional minor copy number (mCN) aberrations and mutation data, their performance improved. The fourth model, which included both SNV and CNA features, demonstrated a higher AUC compared to the first and third models, which contained only SNV or CNA features. The fifth model, which incorporated all features and information, achieved the highest AUC value, making it the optimal model in terms of performance. Figure S5 displays the boxplots of predicted HRD probabilities using LOOCV among HRD+ and HRD− groups.

Once the performance using internal validation was evaluated, we retrained our five models using all observations (Fig. S6) and computed the importance score for each feature (Fig. 3B). The simplest model (CNA) depended heavily on copy number signatures CX1 and CX3. This is not surprising, as CX3 has been proposed as a marker for HRD status [30]. When ASCN information was incorporated, the scarHRD score emerged as the most important feature in the model, increasing the AUC by 3.6%. Although models that combined several data types tended to rely less on individual features, it was still the most important feature in the model. Other relevant features in the models were SBS3 and ID6, markers of HRD; SBS30, a marker of deficiency in base excision repair (BER), possibly not compatible with the HRD phenotype; and ASCN signature CN17, associated with HRD.

Given that HRDetect utilises structural variant information, which is not present in our classifiers, we compared the correlation between the six features used by that classifier (microhomology‐mediated deletions, HRD index, base substitution signature 3, base substitution signature 8, rearrangement signature 3 and rearrangement signature 5), and the features used in our models. As HRDetect requires WGS, we performed this comparison only on the ICGC dataset (Fig. 3C). We were interested in identifying whether the most complex features that require WGS (microhomology‐mediated deletions, rearrangement signature 3 and rearrangement signature 5) were captured by any of our features. Notably, microhomology‐mediated deletions exhibited a high correlation with the indel signatures ID6 and ID8. This finding is unsurprising, as the spectra for ID6 and ID8 predominantly consist of deletions involving microhomology. We also identified statistically significant correlations between the two structural variant features and several of the copy number‐based features, particularly the scarHRD score.

Of note, we observed a high correlation between base substitution signature 3 from HRDetect and our SBS3, with a correlation coefficient of 0.63 and a *P*‐value <0.001. Although they represented the same feature, they stemmed from different SBS signature versions, with ours derived from the latest cosmic version 3.3 rather than the previous cosmic version 2. This somewhat low correlation is due to different data transformation processes, with a correlation of up to 0.953 before the transformation. Figure S7 shows the Pearson's product moment correlation coefficient between the six features of HRDetect and selected features of three blocks in the external validation set from Genomics England [36] (GEL), showing similar conclusions. Finally, Fig. S8 presents the Pearson's product moment correlation coefficient between our whole set of features.

We also evaluated the predictive ability of our ASCN features compared to the performance of scarHRD, which is known to be a strong predictor. We fitted an additional model, no_scarHRD, with all the features from model 2 except the scarHRD score and compared its performance with that of scarHRD. Figure 3A shows a better performance of the no_scarHRD model in terms of AUC than the CNA model, indicating that copy number signatures derived from ASCN provide additional information that enhances HRD prediction. Comparing directly our ASCN model with scarHRD, we observed a lower AUC in the combined dataset, but a higher AUCPR (Fig. S4a).

Although the true HRD labels are entirely defined based on *BRCA1/2* status, our semi‐supervised approach may have the potential to identify additional HRD+ samples that arise from other mechanisms. We hypothesised that these newly identified HRD+ samples, predicted by both our model and the scarHRD method, would share genomic features similar to those of true HRD+ samples (as defined by *BRCA1/2* status). To investigate whether the genomic features of samples labelled as true HRD− (based on *BRCA1/2* status) but classified as HRD+ by our model and the scarHRD method resemble those of true HRD+ samples, we performed a principal component analysis across all features. Figure S4b visualises the first two principal components (PC1 and PC2) based on samples from the combined cohorts, excluding those with unknown HRD status. Figure S4b reveals that samples predicted as HRD+ but labelled as HRD‐ (false positives) cluster with true HRD+ samples. Similar results were also observed in the predictions of the scarHRD method. These findings suggest that both our model and the scarHRD method can identify HRD+ samples that cannot be classified as such based solely on *BRCA1/2* status but may exhibit HRD due to other underlying factors, and these newly identified HRD+ samples exhibit genomic features similar to *BRCA1/2*‐deficient samples.

We also examined the relationship between scarHRD scores and predicted probabilities from our fifth model by stratifying samples based on their true labels and comparing their scores. Figure S4c shows a statistically significant difference in scarHRD scores between samples predicted as HRD+ and HRD− by our model. Figure S4d shows a significant correlation between the two scores.

Furthermore, the recommended threshold of 42 for scarHRD may lead to a higher false positive rate, as these analyses indicate. This value was based on the likelihood of response to platinum‐based chemotherapy [16]. However, our findings indicate that this threshold may need reassessment based on specific dataset distributions or clinical applications, although such analysis is beyond the scope of this study.

In summary, we developed a series of models of increasing complexity that integrate features from different data types and can learn from both *BRCA1/2* mutant/wild‐type samples as well as samples with unknown HRD status.

### 3.1 External validation

**Fig. 4:**
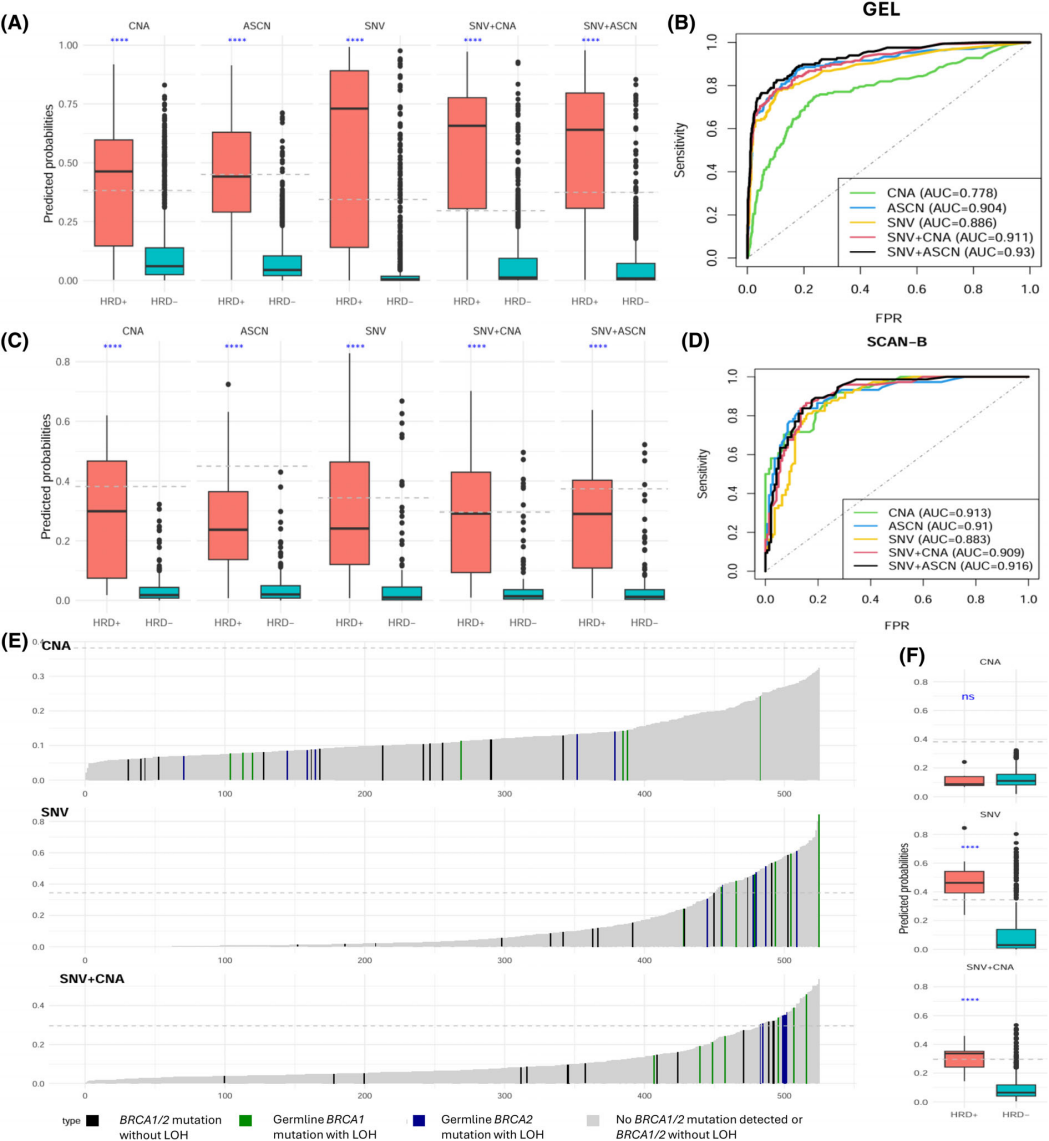
(A) Box plots of predicted probabilities for five models in HRD+ and HRD− groups in the GEL dataset (Wilcoxon rank‐sum test, ****P < 0.0001. The dashed line indicates the optimal threshold. The whiskers represent the range [Q1–1.5 × IQR, Q3 + 1.5 × IQR], where Q1 is 25th percentile, Q3 is 75th percentile, and IQR = Q3 22 Q1.). (B) Receiver Operating Characteristic (ROC) curves for the predicted probabilities on the five models based on the HRD status in the GEL dataset. (C). Box plots of predicted probabilities for five models in HRD+ and HRD− groups in the SCAN‐B dataset (Wilcoxon rank‐sum test, ****P < 0.0001. The dashed line indicates the optimal threshold. The whiskers represent the range [Q1–1.5 × IQR, Q3 + 1.5 × IQR], where Q1 is 25th percentile, Q3 is 75th percentile, and IQR = Q3 − Q1.). (D) ROC curves for the predicted probabilities on the five models based on the HRD status in the SCAN‐B dataset. (E) Predicted probabilities derived from three models in the MyBrCa dataset, sorted from smallest to largest and coloured by HRD status. Unknown‐status cases are represented in black, HRD− cases in light grey, and HRD+ cases in green and blue. The dashed line represents the optimal threshold. (F) Box plots of predicted probabilities derived from three models in HRD+ and HRD− groups in the MyBrCa dataset (Wilcoxon rank‐sum test, “ns” = non‐significant, ****P < 0.0001. The dashed line indicates the optimal threshold. The whiskers represent the range [Q1–1.5 × IQR, Q3 + 1.5 × IQR], where Q1 is 25th percentile, Q3 is 75th percentile, and IQR = Q3 − Q1.).

We validated our models on six different datasets (see Table S1). Table S4 provides the predicted HRD probabilities for all cohorts and models. Figure 4A illustrates the results for the GEL (Genomics England Breast Cancer [36], *n* = 2964) dataset. All models demonstrated good performance in identifying *BRCA1/2*‐deficient tumours; and as seen in the training dataset, models that integrated several data types produced better performance, proving that augmenting information improves model prediction. Figure 4B illustrates the AUC values, increasing from 0.778 for the model relying solely on CNA data to 0.930 in the full model. Additionally, we conducted a comparative analysis of our models against established methodologies, including scarHRD, HRDetect and CHORD, using the GEL dataset. Table 1 shows the confusion matrices comparing the *BRCA1/2* status in the cohort and the predictions derived from all models considered. While the scarHRD method showed the highest AUC value, it produced a larger proportion of false positives (FP), resulting in diminished precision. Conversely, the WGS‐based HRDetect and CHORD methodologies exhibited comparable performances, characterised by relatively lower AUC values and a propensity for false negatives (FN), consequently affecting sensitivity adversely. Notably, our models consistently improved in performance with the integration of additional information and exhibited comparable or even superior performance compared to other methods, attaining a good balance between sensitivity and specificity. Similar results can be seen in Fig. 4C,D for the SCAN‐B (Sweden Cancerome Analysis Network–Breast [37, 38], *n* = 231) dataset. We also predicted HR status in the MyBrCa dataset (The Malaysian Breast Cancer study [40], *n* = 525). The results for models 1, 3, and 4 (given the absence of ASCN data) are shown in Fig. 4E,F. Figure 4E shows the predicted probabilities derived from these three models, and Fig. 4F compares predicted probabilities in HRD− and HRD+ tumours. Both figures highlight that in the MyBrCa dataset, the models that include SNV features discriminate much better HRD+ cases.

**Table 1:** Confusion matrices for the self‐training approach across five models, scarHRD, HRDetect and CHORD (Classifier of HOmologous Recombination Deficiency) methods in the GEL cohort. AUC, area under the curve; AUCPR, area under the precision‐recall curve; MCC, Matthews correlation coefficient.

Method	Prediction	HRD+		HRD−		HRD unknown		AUC	AUCPR	Sensitivity	Specificity	Precision	MCC
CNA	Yes	98	59.04%	149	14.19%	552	31.58%	0.778	0.412	0.590	0.858	0.397	0.383
	No	68	40.96%	901	85.81%	1196	68.42%						
ASCN	Yes	81	48.80%	17	1.62%	243	13.90%	0.904	0.728	0.488	0.984	0.827	0.595
	No	85	51.20%	1033	98.38%	1505	86.10%						
SNV	Yes	108	65.06%	64	6.10%	328	18.76%	0.886	0.694	0.651	0.939	0.628	0.581
	No	58	34.94%	986	93.90%	1420	81.24%						
SNV + CNA	Yes	125	75.30%	92	8.76%	454	25.97%	0.911	0.732	0.753	0.912	0.576	0.597
	No	41	24.70%	958	91.24%	1294	74.03%						
SNV + ASCN	Yes	117	70.48%	33	3.14%	367	21.00%	0.930	0.783	0.705	0.969	0.780	0.703
	No	49	29.52%	1017	96.86%	1381	79.00%						
scarHRD	Yes	124	74.70%	82	7.81%	747	42.73%	0.940	0.752	0.747	0.922	0.602	0.612
	No	42	25.30%	968	92.19%	1001	57.27%						
HRDetect	Yes	93	56.02%	22	2.10%	211	12.07%	0.895	0.741	0.560	0.979	0.809	0.633
	No	73	43.98%	1028	97.90%	1537	87.93%						
CHORD	Yes	87	52.41%	14	1.33%	179	10.24%	0.840	0.681	0.544	0.986	0.861	0.649
	No	73	43.98%	1008	96.00%	1508	86.27%						
	NA	6	3.61%	28	2.67%	61	3.49%						

**Fig. 5:**
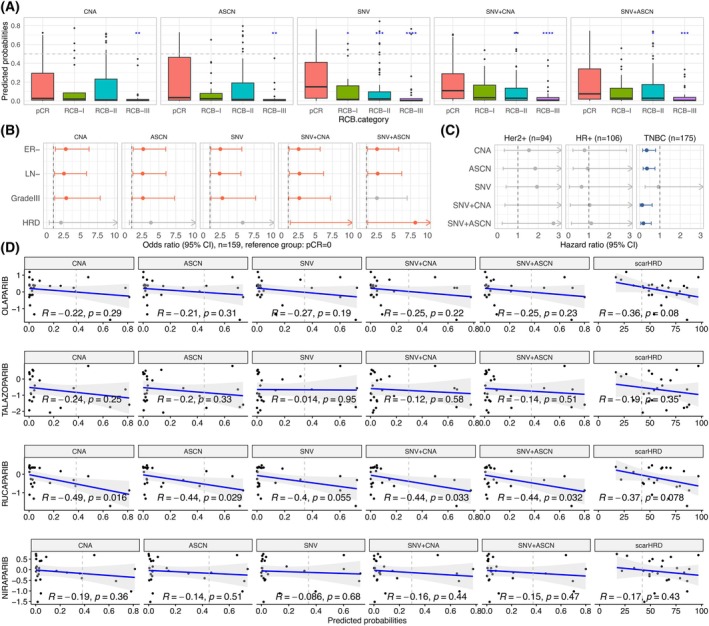
(A). Box plots showing monotonic association between residual cancer burden (RCB) classes and predicted probabilities obtained through validation for the five models in the TransNEO dataset. (*P*‐values = 0.031, 0.005, 0.003, 0.001 and 0.001 for five models respectively based on ordinal logistic regression; Wilcoxon rank‐sum tests with pathological complete response (pCR) as the reference group; all *P*‐values are two‐sided; *P < 0.05, ***P < 0.001, ****P < 0.0001; RCB‐I, RCB‐II and RCB‐III reflect the extent of residual cancer burden according to the RECIST criteria. The dashed line indicates the optimal threshold. The whiskers represent the range [Q1–1.5 × IQR, Q3 + 1.5 × IQR], where Q1 is 25th percentile, Q3 is 75th percentile, and IQR = Q3 − Q1.). (B) The odds ratio of the predicted HRD probabilities for five multivariable logistic models. The variables included are Oestrogen receptor (ER) status, lymph nodes (LN) and grades, equivalent to the clinical base model described in the original paper. Dots represent point estimates, and bars represent 95% confidence intervals. (C) Hazard ratios for 10 univariable Cox models predicting relapse‐free survival in patients that received adjuvant chemotherapy in the METABRIC dataset. Sample sizes were 94 for Her2‐positive (Her2+), 106 for ER+/Her2− (HR+), and 175 for ER‐/Her2− (TNBC, Triple‐negative breast cancer) cases. Each model has a unique predictor based on one of our semi‐supervised models. Dots represent point estimates, and bars represent 95% confidence intervals. (D) Scatter plots between predicted HRD probabilities generated by five models and scarHRD scores on 25 breast cancer cell lines from the CCLE dataset and their sensitivity to PARP inhibitors as assessed through the PRISM metric. Pearson correlation coefficients and *P*‐values are shown. The vertical dashed lines represent the optimal thresholds for the five models, as well as the scarHRD score threshold of 42.

Next, we assessed the response of patients with HRD to neoadjuvant chemotherapy using the TransNEO [39] dataset (*n* = 164). This dataset includes 164 patients with specific chemotherapy regimens based on block‐sequential taxane and anthracycline chemotherapy. Treatment response was assessed with the residual cancer burden (RCB) criteria at the time of surgery. Figure 5A demonstrates a significant and roughly monotonic association between the RCB class and predicted probabilities for the five models. Figure 5B presents the odds ratio of the predicted HRD probabilities for five multivariable logistic models. The variables included are ER status, lymph nodes, and grades, equivalent to the clinical base model described in the original paper [39]. The HRD probabilities generated by the models containing both CNA and SNV were significantly associated with pathological complete response (pCR). The inclusion of SNV features greatly improved the performance in identifying HRD+ tumours in this dataset (Fig. S9a). Similar to the MyBrCa dataset, this observation suggests a substantial enhancement in classification accuracy attributed to SNV features.

To assess the association between our HRD status predictions and adjuvant chemotherapy response, we focused on a subgroup consisting of 375 individuals who underwent chemotherapy in the METABRIC [41, 42, 43] dataset. Among these 375 patients, we classified them into three molecular subtypes based on ER and Her2 status and denoted them as Her2+, ER+/Her2− (HR+) and ER‐/Her2− (TNBC), with sample sizes of 94, 106, and 175, respectively. Using endpoint relapse‐free survival (RFS), we fitted five independent univariable Cox models for every group separately, using the probabilities of HRD provided by each of our semi‐supervised models. Figure 5C illustrates that, in the TNBC subgroup, HRD probabilities were prognostic except for the SNV model. This was attributed to the use of a targeted panel sequencing for SNV data in the METABRIC dataset, which sequenced only 173 genes, limiting the ability to call mutational signatures with such a small gene panel. Interestingly, the HRD status was not prognostic in the Her2+ and HR+ subgroups, although we note that in METABRIC none of the Her2+ patients received targeted therapy.

To evaluate the association between our HRD status predictions and sensitivity to PARP inhibitors, we leveraged genomic data from breast cancer cell lines and PARP inhibitor sensitivity profiles sourced from the CCLE dataset (Cancer Cell Line Encyclopaedia [44], *n* = 25). Figure 5D displays the correlation between the predicted HRD probabilities for 25 breast cancer cell lines, as generated by our models, and their sensitivity to PARP inhibitors, assessed through PRISM metrics [45]. Predicted HRD probabilities exhibited a negative correlation with drug response, indicating that HRD status predicted sensitivity to PARP inhibitors, particularly Rucaparib, in models incorporating SNV features. However, we did not observe a statistically significant correlation with the other three types of PARP inhibitors. Similar results were obtained with scarHRD (Fig. 5D). We also investigated the association between our HRD status predictions and sensitivity to several platinum‐based chemotherapies in the 25 breast cancer cell lines with complete PRISM data (Fig. S9b). We also found a negative correlation, especially in the case of Carboplatin and Nedaplatin, indicating that the predicted HRD status is also associated with sensitivity to these compounds.

Finally, Fig. S10 exhibits the characteristics of tumours stratified by predicted HRD status. Table S6 shows the proportions of predicted HRD status across clinical subtypes in all cohorts, and Table S7 provides the *P*‐values obtained from Pearson's Chi‐squared test, assessing the association between clinical information and predicted HRD status across various cohorts. Similarly to the results obtained with the training cohorts and shown in Fig. 1, HRD+ tumours typically show characteristics such as being ER‐negative, PR‐negative, higher tumour grades and a predominant presence in the basal‐like and IntClust 10 subtypes, as well as a younger age at diagnosis. The subset of the SCAN‐B dataset with DNA sequencing exclusively encompasses triple‐negative breast cancers; therefore, their characteristics differ from those of the other cohorts. Table S8 shows the proportions of predicted HRD status in triple‐negative breast cancer (TNBC) and ER‐positive samples based on the fifth model across various cohorts. As expected, the proportion of samples predicted to be HRD+ is significantly higher in TNBC samples compared to ER‐positive samples. However, these numbers differ across cohorts.

### 3.2 Somatic mutational profiles of HRD+ tumours

**Fig. 6:**
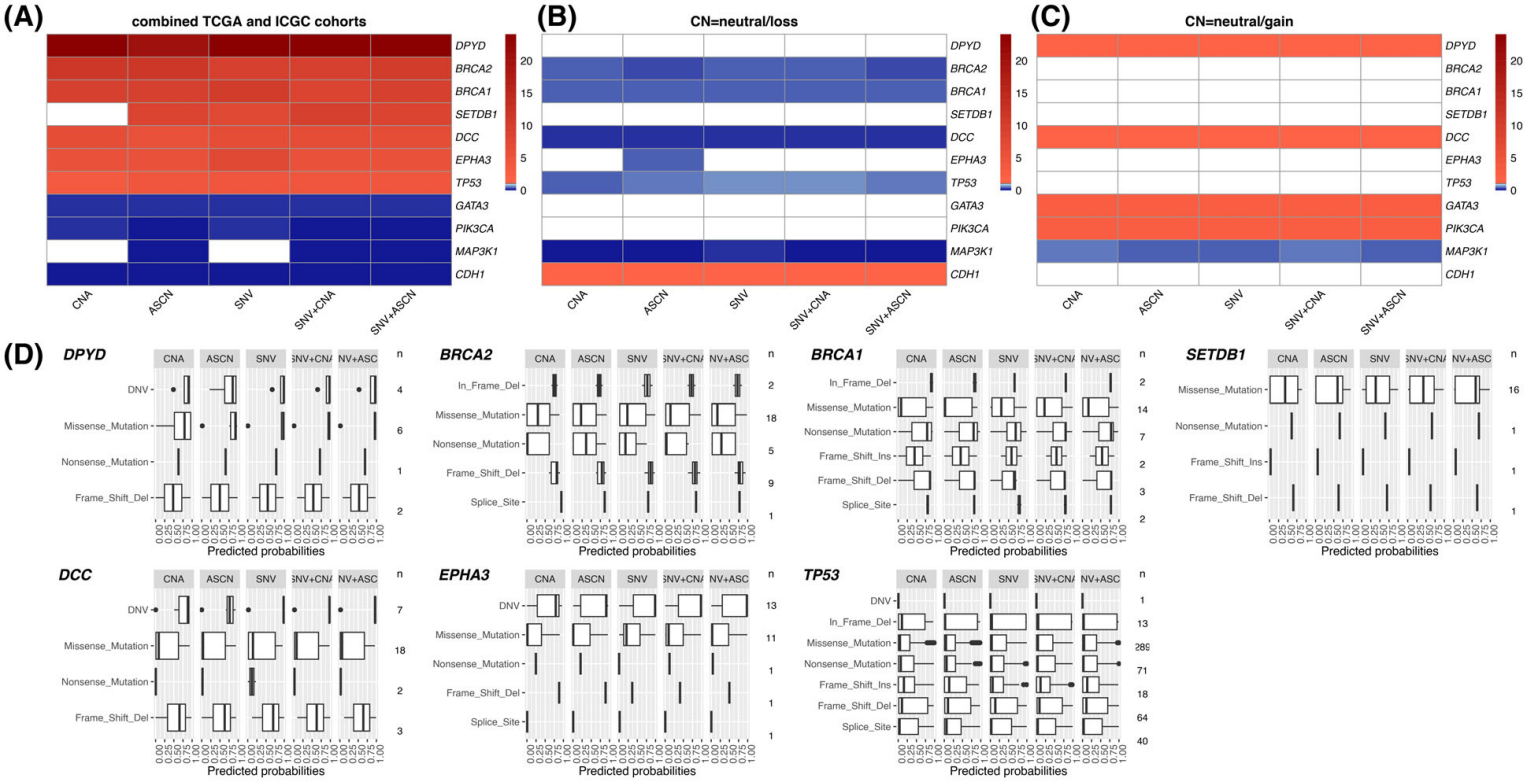
(A) Heatmaps with odds ratios from Fisher's exact test to detect associations between somatic mutations and predicted HRD status derived from our five models within the combined TCGA and ICGC cohorts. Significant odds ratios (OR), considered as adjusted *P*‐values using Hochberg's multiple testing correction method, are highlighted in red (OR >1) or blue (OR <1). Non‐significant cases are shown in white. (B, C) Heatmaps of odds ratios from Fisher's exact test showing associations between copy number states of selected 11 genes (B: neutral versus loss; C: neutral versus gain) and predicted HRD status derived from our five models within the combined TCGA and ICGC cohorts. Significant odds ratios (OR) are highlighted in red (OR >1) or blue (OR <1). Non‐significant cases are shown in white. (D) Box plots of predicted HRD probabilities generated by our models for different variant types in the combined dataset for seven selected genes. The whiskers represent the range [Q1–1.5 × IQR, Q3 + 1.5 × IQR], where Q1 is 25th percentile, Q3 is 75th percentile, and IQR = Q3 − Q1. DNV, double nucleotide variants.

Using a curated list of 781 genes related to cancer and the homologous recombination pathway [21], we employed Fisher's exact test to assess the association between the presence of a somatic mutation in these genes and the predicted HRD status among the five models in our training cohorts. Figure 6A shows that, beyond *BRCA1/2*, this analysis also identified nine other noteworthy genes, which were *DPYD*, *SETDB1*, *DCC*, *EPHA3*, *TP53*, *GATA3*, *PIK3CA*, *MAP3K1* and *CDH1*. Among these, *DPYD*, *BRCA2*, *BRCA1*, *SETDB1*, *DCC*, *EPHA3* and *TP53* mutations were significantly enriched in HRD+ tumours. As expected, *BRCA1/2* somatic status was associated with HRD+. *DPYD* encodes dihydropyrimidine dehydrogenase (*DPD*), an enzyme critical to the metabolism of chemotherapeutic drugs, notably 5‐fluorouracil (5‐FU), which is widely used in the treatment of various cancers. Alterations in the *DPYD* gene can significantly influence the efficacy and safety of 5‐FU‐based therapy [53, 54]. Such mutations may lead to an aberrant metabolism of 5‐FU, affecting the drug's pharmacokinetics and potentially altering treatment outcomes for patients with HRD. However, there were only 13 variants in this gene among the two studies. *SETDB1* (SET Domain Bifurcated 1) occupies a pivotal position in the intricate regulation of chromatin architecture and gene expression. It serves as an additional compacting factor in promoting homologous recombination, thereby ensuring the faithful completion of this crucial DNA repair mechanism [55]. The potential interplay between mutations in *SETDB1* and HRD may be attributed to their impacts on gene expression dynamics and the regulation of the cell cycle. *DCC* (Deleted in Colorectal Carcinoma) is an important tumour suppressor gene, whose expression is significantly reduced or absent in most advanced colorectal cancers and many other cancers [56, 57]. *DCC* expression is closely linked to DNA damage response and repair (DDR) pathways. Samples with *DCC* mutations are often enriched in DDR pathways, highlighting its critical role in DNA damage repair [58]. Inactivation of *DCC* impairs repair signalling, resulting in unrepaired DNA errors and genomic instability. Genomic instability is a hallmark of homologous recombination deficiency (HRD); thus, the loss or mutation of *DCC* may promote HRD by exacerbating genomic instability. *EPHA3* (Ephrin type‐A receptor 3) is a receptor tyrosine kinase that plays a crucial role in regulating cell behaviour. *TP53*, the most mutated tumour suppressor in breast cancer, regulates cell cycle progression and DNA damage repair [59, 60]. HRD+ tumours may require inactivation of *TP53* to avoid cell cycle arrest at G2/M post DNA damage [61, 62].

On the other hand, *GATA3*, *PIK3CA*, *MAP3K1* and *CDH1* mutations were associated with HR proficiency. Given that HRD‐negative tumours were mostly ER+ (Fig. 1), we could probably infer that those genes were just highlighting the fact that they were commonly mutated in ER+ tumours. Figure S11 and Table S9 show the proportion of somatic mutations in these genes in the training cohorts. In addition, Table S9 also displays the odds ratios from Fisher's exact test to detect associations between somatic mutations and predicted HRD status.

We further explored the relative pathogenicity of different variant types using the combined dataset of the two cohorts, as depicted in Fig. 6D. As expected, somatic truncating mutations in *BRCA1* and *BRCA2* were associated with higher predicted HRD probability. However, missense or in‐frame variants were sometimes related to HRD, in particular *DPYD* and *TP53*. Consequently, it is challenging to discern which mutation types confer greater pathogenicity and an increased probability of the HRD phenotype.

Additionally, we investigated the relationship between copy number aberrations of these 781 genes and the predicted HRD status. Tables S10 and S11 show the results of this analysis (odds ratios and FDR adjusted *P*‐values) for gains and losses. Based on the predictions of our ASCN+SNV model, we identified 99 genes where the proportion of gains was higher in the HRD+ predicted samples, with interesting examples such as *GATA3* or *PIK3CA* and 361 genes where the proportion of losses was higher, including well‐known tumour suppressors such as *BRCA2*, *BRCA1*, *TP53* and *MAP3K1*, revealing the importance of LOH events paired with mutations.

## 4 Discussion

The clinical application of useful biomarkers is often restricted to specific assays, techniques or data types. Consequently, the availability or affordability of these tests in a clinical context may limit the benefit of specific treatments in certain patients. In this study, we have explored the feasibility of estimating a biomarker using different data types. We have focused on detecting HRD+ tumours in breast cancer using data obtained from different DNA assays, as specific treatments are beneficial for this subgroup of patients.

Although there are already several good options to estimate HRD using DNA (HRDetect [20], scarHRD [16]) or RNA‐based signatures [31, 32], a systematic comparison and independent validation over large cohorts has not been performed to date. We have compared performance and correlated different features across methods and data types. Overall, our analysis highlights that it is possible to adapt a tailor‐made strategy to derive a HRD predictor to specific resources in terms of analytical ability and sequencing budget in a clinical context to reach a given sensitivity and specificity. The performance of our methods in external datasets shows that whole genome sequencing and the integration of several data types improves HRD+ tumour detection, as expected. The quality of the features derived with a smaller amount of information and the performance of the simplest models are more dependent on the bioinformatic algorithms employed to derive those features. Our work shows, however, that good performance can be achieved with affordable sequencing strategies, although more work must be done in the pre‐processing steps to call these features, possibly with the use of specific training data to ensure optimisation of data acquisition, normalisation and biomarker estimation. Although it is difficult to derive specific guidelines, we recommend at least WES to obtain good performance in models 3–5, as they require accurate estimation of mutational signatures. We have shown that data derived from targeted sequencing may produce low‐quality estimation of SBS and indel signatures in METABRIC, with only 173 genes sequenced. Any technology that can produce good quality ASCN data will be adequate for model 2, as in the cases illustrated with Affymetrix SNP 6 or WES. For data obtained with shallow whole genome sequencing, we only recommend using our simplest model 1, but we note that copy number signatures rely on certain copy number call methods. As the goal of this study was not to derive a definite model for HRD prediction, we did not try to optimise our models by pre‐processing each dataset with the same tools. On the contrary, we used available data produced and normalised by different teams using different tools, as we wanted to prioritise feature robustness. This comes with some disadvantages, as in the case of models 3–5 in METABRIC that highlight the limitations in feature estimation.

Our models have shown a good balance of sensitivity and specificity in the external datasets based on a fixed threshold for HRD classification. Future studies could explore adaptive thresholding strategies based on cohort‐specific probability distributions. This is particularly important in the context of patient allocation to treatment, as false positives may undergo unnecessary treatments that incur additional costs and provide no clinical benefit, exposing patients to potential side effects. On the other hand, false negatives may result in patients missing out on effective treatments, potentially leading to disease progression and poorer outcomes.

## 5 Conclusions

We have used *BRCA1/2* mutational status as the gold standard for HRD labelling. Although there are other mechanisms that can produce the phenotype, such as structural variants in *BRCA1/2* or mutations in other related genes, the genomic profile should be similar, and our selection of features and the use of unlabelled samples should capture these alternative mechanisms. This means that the number of false positives might be smaller than what we have reported, and it makes difficult the assessment and comparison of different methods. In the GEL dataset, we have classified as HRD+ 17.4% of the cases, compared to only 5.6% *BRCA1/2*‐deficient tumours. Unfortunately, it is very difficult to quantify the percentage of extra patients who would benefit from HRD‐targeted treatments, and the number of cell lines is not large enough to answer that question. Still, we hope that our analytical findings will inspire advancements in current clinical practices for HRD detection, enabling more accurate predictions at a reduced cost and facilitating personalised treatment plans for HRD‐positive patients.

## Conflict of interest

The authors declare no conflict of interests.

## Author contributions

RZ and OMR conceived the study, led data analysis, and wrote the manuscript. KE and S‐FC interpreted results and guided the data analysis. RMG and MC provided bioinformatics support. RM, SM, and S‐JS provided statistical advice and expertise. JWP and SHT contributed data from the Malaysian Breast Cancer study for validation and provided expertise. CC and PAWE provided pathology and clinical expertise. All authors contributed comments, read, and approved the manuscript.

## Supporting information


**Fig. S1.** A summary of the five trained models categorised by the features they incorporate.
**Fig. S2.** Boxplots and Wilcoxon rank‐sum test results (‘ns’ = non‐significant, *P < 0.05, **P < 0.01, ***P < 0.001, ****P < 0.0001) of all features from three blocks in combined datasets for HRD status.
**Fig. S3.** Receiver Operating Characteristic (ROC) curves for Leave‐one‐out cross‐validation (LOOCV) predicted probabilities across five models based on the HRD status within the combined cohorts and each individual cohort. The first row displays results from our random forest‐based self‐training methods (same as Figure 3a), while the second and third rows show results from gradient boosting and logistic regression‐based self‐training methods, respectively.
**Fig. S4.** (a) Precision‐Recall (PR) curves for Leave‐one‐out cross‐validation (LOOCV) predicted probabilities in seven models based on the HRD status within the combined two cohorts and each cohort. The no_scarHRD model incorporates relative copy number features and ASCN features while excluding the scarHRD score, whereas the scarHRD model consists solely of the scarHRD score. The dashed baseline is determined by the ratio of HRD positives (P) and negatives (N) as y = P/(P + N). (b) Principal component analysis (PCA) visualisation of the first two principal components (PC1 and PC2) based on samples from the combined cohorts, excluding those with unknown HRD status. Data points are distinguished by shape based on their true HRD status, and colour‐coded according to their predicted HRD status, derived from our fifth model and the scarHRD method, shown separately. Ellipses represent the two clusters formed according to the true HRD status. (c) Boxplots and Wilcoxon rank‐sum test results (****P < 0.0001) comparing scarHRD scores between the two HRD categories predicted by our fifth model among samples with true HRD+ and true HRD− in the combined cohorts, shown separately. The horizontal dashed line represents the scarHRD score threshold of 42. (d) Scatter plots of predicted HRD probabilities from our fifth model versus scarHRD scores for samples with true HRD+ and true HRD− labels in the combined cohorts, shown separately. Pearson's correlation coefficients and *P*‐values are provided. The horizontal dashed line represents the scarHRD score threshold of 42, while the vertical dashed line indicates the classification threshold of 0.374 for our fifth model.
**Fig. S5.** Boxplots of predicted HRD probabilities computed using Leave‐one‐out cross‐validation (LOOCV) among HRD+ and HRD− groups in the combined and two training cohorts separately (Wilcoxon rank‐sum test, ****P < 0.0001; the dashed line represents the optimal threshold.).
**Fig. S6.** Predicted probabilities for the training observations in the combined dataset in the five models, sorted from smallest to largest and coloured by HRD status. Unlabelled cases are represented in black, HRD− cases in light grey, and HRD+ cases in various colours. The dashed line represents the optimal threshold.
**Fig. S7.** Pearson's product moment correlation coefficient between six features of HRDetect and selected features of three blocks in the GEL set, where the non‐significant results are blank.
**Fig. S8.** Pearson's product–moment correlation coefficient between the selected features among and within the three blocks in the combined dataset, where the non‐significant results are blank. (a) shows the correlation between features in CNA and SNV features, (b) between ASCN and SNV features, (c) between CNA and ASCN features, (d) between ASCN features, (e) between SNV features, and (f) between CNA features.
**Fig. S9.** (a) Boxplots of predicted HRD probabilities for five models among HRD+ and HRD− groups in TransNEO (Wilcoxon rank‐sum test, ‘ns’ = non‐significant, **P < 0.01, ***P < 0.001; dashed line is the optimal threshold). (b) Scatter plots between predicted HRD probabilities generated by five models and scarHRD scores on 25 breast cancer cell lines from the CCLE dataset and their sensitivity to different platinum‐based chemotherapies as assessed through the Profiling relative inhibition simultaneously in mixtures (PRISM) metric. Pearson's correlation coefficients and *P*‐values are shown. The vertical dashed lines represent the optimal thresholds for the five models, as well as the scarHRD score threshold of 42.
**Fig. S10.** Characteristics of tumours stratified based on predicted HRD‐positive (HRD+) and HRD‐negative (HRD−) status across five models in TCGA, ICGC, METABRIC, GEL, SCAN‐B, TransNEO and MyBrCa (Models 1, 3 and 4). Note that the SCAN‐B cohort only contains triple negative tumours.
**Fig. S11.** Proportions of somatic mutations in tumours stratified based on the *BRCA1/2* status (true label) and the predicted HRD‐positive (HRD+) and HRD‐negative (HRD−) status across five models in the combined TCGA and ICGC cohorts.

**Table S1.** Basic information, sample sizes and HRD status in all the cohorts.
**Table S2.** Features in each data type block.
**Table S3.** List of all the five models depending on features used.
**Table S4.** Predicted HRD probabilities in five models for all cohorts.
**Table S5.** Hazard ratios (HR) for five univariable Cox models predicting relapse‐free survival in patients that received adjuvant chemotherapy in the METABRIC dataset, where the five variables are age (both continuous and dichotomised at 50), lymph node (LN) status, tumour stage and tumour grade.
**Table S6.** Proportions of predicted HRD status across clinical subtypes in all cohorts, corresponding to Fig. S10.
**Table S7.** *P*‐values from Pearson's Chi‐squared test to assess the correlation between clinical information and HRD+/− status in different cohorts.
**Table S8.** Proportions of predicted HRD status in triple‐negative breast cancer (TNBC) and ER‐positive samples based on the fifth model across various cohorts.
**Table S9.** Odds ratios from Fisher's exact test to detect associations between somatic mutations and predicted HRD status, and proportions of somatic mutations in tumours stratified based on the *BRCA1/2* status (true label) and the predicted HRD‐positive (HRD+) and HRD‐negative (HRD−) status across five models in the combined TCGA and ICGC cohorts, corresponding to Fig. S11.
**Table S10.** Odds ratios from Fisher's exact test to detect associations between copy number gains and predicted HRD status across five models in the combined TCGA and ICGC cohorts. *P*‐values are adjusted for multiple testing using FDR (Benjamini‐Hochberg correction).
**Table S11.** Odds ratios from Fisher's exact test to detect associations between copy number losses and predicted HRD status across five models in the combined TCGA and ICGC cohorts. *P*‐values are adjusted for multiple testing using FDR (Benjamini‐Hochberg correction).

## Data Availability

The publicly available datasets used in this study can be accessed as follows: METABRIC [41, 42, 43] and TCGA [34] data are available via the Genomic Data Commons Data Portal (https://portal.gdc.cancer.gov/) and cBioportal (https://www.cbioportal.org/), ICGC [35] data are available from https://dcc.icgc.org, and SCAN‐B [37, 38] data are available from https://data.mendeley.com/datasets/2mn4ctdpxp/3. TransNEO [39] data have been deposited at the European Genome‐Phenome Archive (EGA) under accession number EGAS00001004582. Access to controlled MyBrCa [40] data will require the approval of the MyBrCa Tumour Genomics Data Access Committee upon request to Soo‐Hwang Teo at genetics@cancerresearch.my. CCLE [44, 45] data are available from https://depmap.org/portal/. With respect to GEL [36] data, research on the de‐identified patient data used in this publication can be carried out in the Genomics England Research Environment subject to a collaborative agreement that adheres to patient‐led governance. All interested readers will be able to access the data in the same manner that the authors accessed the data. For more information about accessing the data, interested readers may contact research-network@genomicsengland.co.uk or access the relevant information on the Genomics England website: https://www.genomicsengland.co.uk/research. The code to run our method, ssc‐HRD (semi‐supervised classifier of HRD) and all analyses is deposited in https://github.com/RONG‐ZHU‐1/ssc‐HRD.
