# Supplementary material for: Detecting homologous recombination deficiency for breast cancer through integrative analysis of genomic data
Source: Mol Oncol. 2025 Apr 22;19(12):3613–33. doi: 10.1002/1878-0261.70041 (PMC12688163; doi:10.1002/1878-0261.70041)

**Detecting homologous recombination deficiency for breast cancer**

**through integrative analysis of genomic data**

Rong Zhu^1,2^, Katherine Eason^3^, Suet-Feung Chin^3^, Paul AW Edwards^4^, Raquel Manzano Garcia^3^, Richard Moulange^2^, Jia Wern Pan^5^, Soo Hwang Teo^5^, Sach Mukherjee^2,6,7^, Maurizio Callari^8^, Carlos Caldas^9^, Stephen-John Sammut^10,11^ and Oscar M Rueda^2*^

*^1^ School of Mathematics and Statistics, Beijing Institute of Technology, Beijing, China*

*^2^ MRC Biostatistics Unit, University of Cambridge, Cambridge, United Kingdom*

*^3^ Cancer Research UK Cambridge Institute, University of Cambridge, United Kingdom*

*^4^ Department of Pathology, University of Cambridge, Cambridge, United Kingdom*

*^5^ Cancer Research Malaysia, No. 1, Jalan SS12/1A, 47500 Subang Jaya, Malaysia*

*^6^ Deutsches Zentrum für Neurodegenerative Erkrankungen (DZNE), Bonn, Germany*

*^7^ University of Bonn, Bonn, Germany*

*^8^ Fondazione Michelangelo, 20121, Milano, Italy*

*^9^ School of Clinical Medicine, University of Cambridge, Cambridge, United Kingdom*

*^10^ Breast Cancer Now Toby Robins Research Centre, The Institute of Cancer Research, London, United Kingdom*

*^11^ The Royal Marsden Hospital NHS Foundation Trust, London, United Kingdom*

*^*^Correspondence: Oscar.Rueda@mrc-bsu.cam.ac.uk*

**Supporting Information**

**Supplementary Figure 1**: A summary of the five trained models categorised by the features they incorporate. (mCN: Minor copy number; LOH: Loss of heterozygosity; TAI: Telomeric allelic imbalance; LST: Large-scale state transitions; SBS: Single base substitution; ID: Insertions and deletions.)


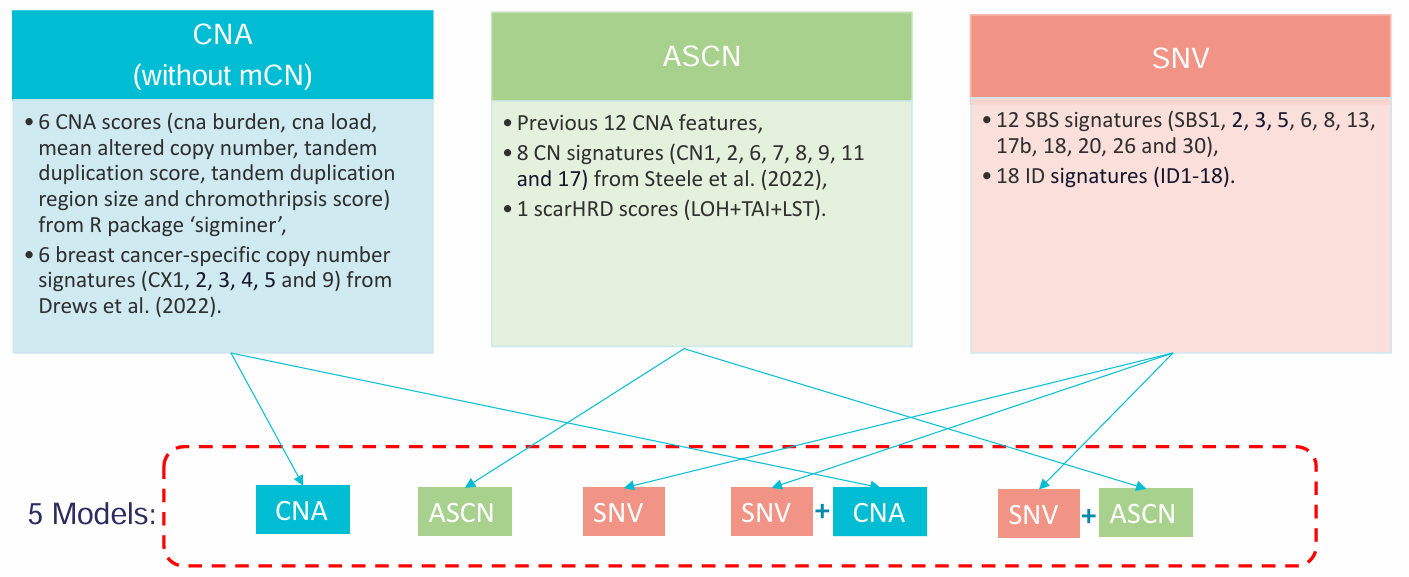


**Supplementary Figure 2**: Boxplots and Wilcoxon rank-sum test results (‘ns’=non-significant, *P < 0.05, **P < 0.01, ***P < 0.001, ****P < 0.0001) of all features from three blocks in combined datasets for HRD status. (MACN: mean altered copy number; TDP: tandem duplication; CN: Copy number; SBS: Single base substitution; ID: Insertions and deletions.)


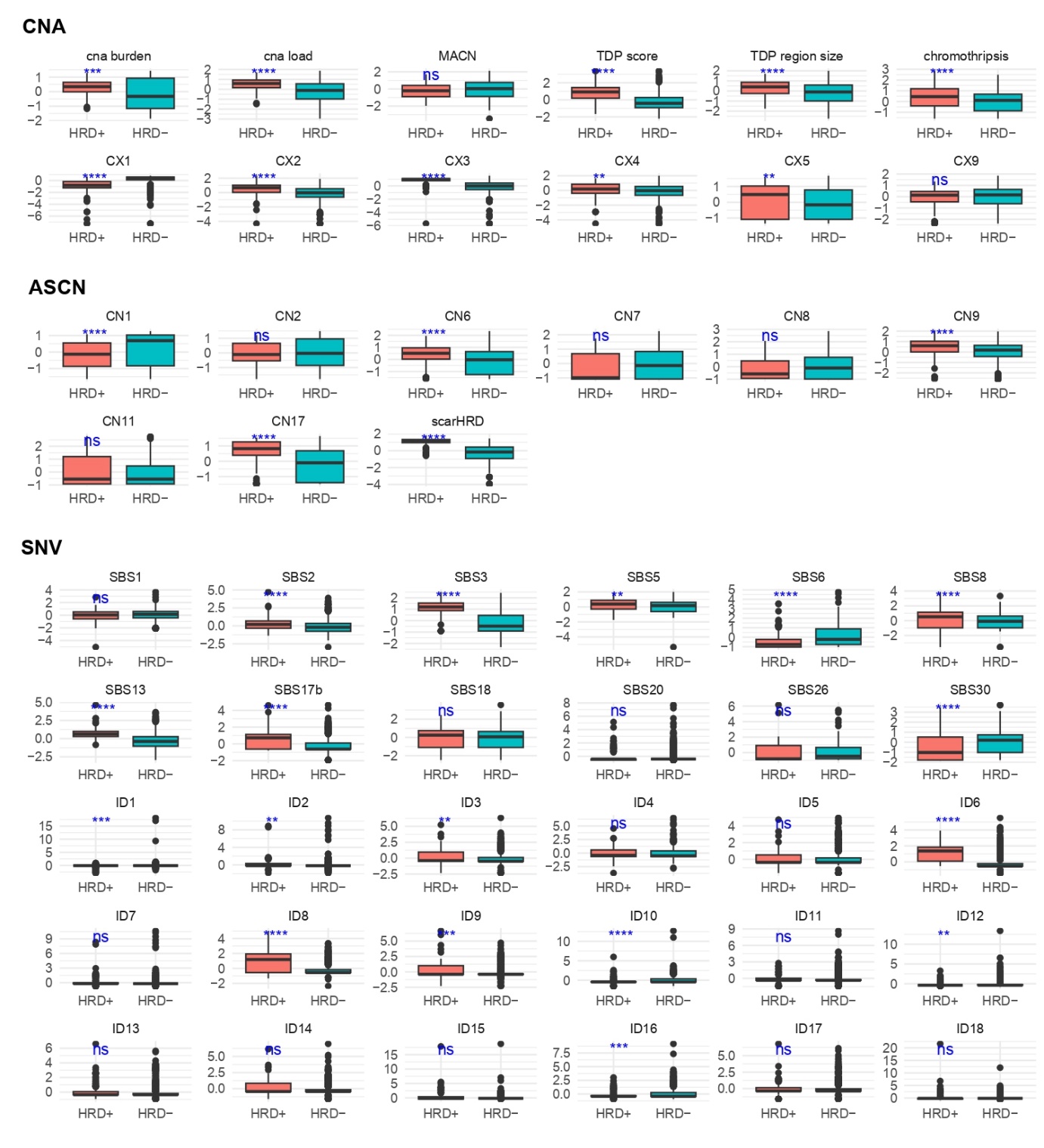


**Supplementary Figure 3**: Receiver Operating Characteristic (ROC) curves for Leave-one-out cross-validation (LOOCV) predicted probabilities across five models based on the HRD status within the combined cohorts and each individual cohort. The first row displays results from our random forest-based self-training methods (same as Figure 3a), while the second and third rows show results from gradient boosting and logistic regression-based self-training methods, respectively.


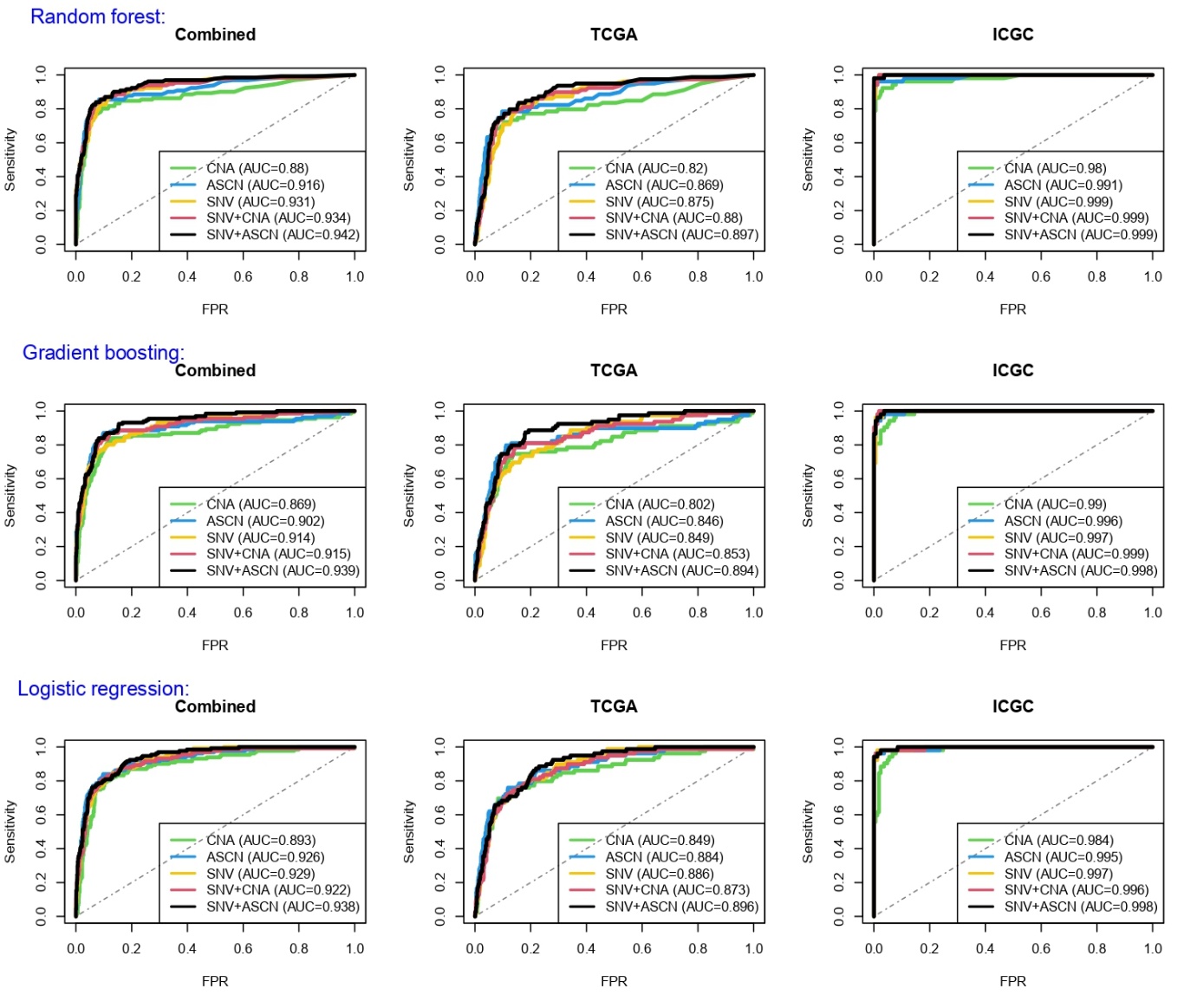


**Supplementary Figure 4**: (a) Precision-Recall (PR) curves for Leave-one-out cross-validation (LOOCV) predicted probabilities in seven models based on the HRD status within the combined two cohorts and each cohort. The no_scarHRD model incorporates relative copy number features and ASCN features while excluding the scarHRD score, whereas the scarHRD model consists solely of the scarHRD score. The dashed baseline is determined by the ratio of HRD positives (P) and negatives (N) as y = P / (P + N). (b) Principal component analysis (PCA) visualization of the first two principal components (PC1 and PC2) based on samples from the combined cohorts, excluding those with unknown HRD status. Data points are distinguished by shape based on their true HRD status, and color-coded according to their predicted HRD status, derived from our fifth model and the scarHRD method, shown separately. Ellipses represent the two clusters formed according to the true HRD status. (c) Boxplots and Wilcoxon rank-sum test results (****P < 0.0001) comparing scarHRD scores between the two HRD categories predicted by our fifth model among samples with true HRD+ and true HRD– in the combined cohorts, shown separately. The horizontal dashed line represents the scarHRD score threshold of 42. (d) Scatter plots of predicted HRD probabilities from our fifth model versus scarHRD scores for samples with true HRD+ and true HRD– labels in the combined cohorts, shown separately. Pearson correlation coefficients and p-values are provided. The horizontal dashed line represents the scarHRD score threshold of 42, while the vertical dashed line indicates the classification threshold of 0.374 for our fifth model.


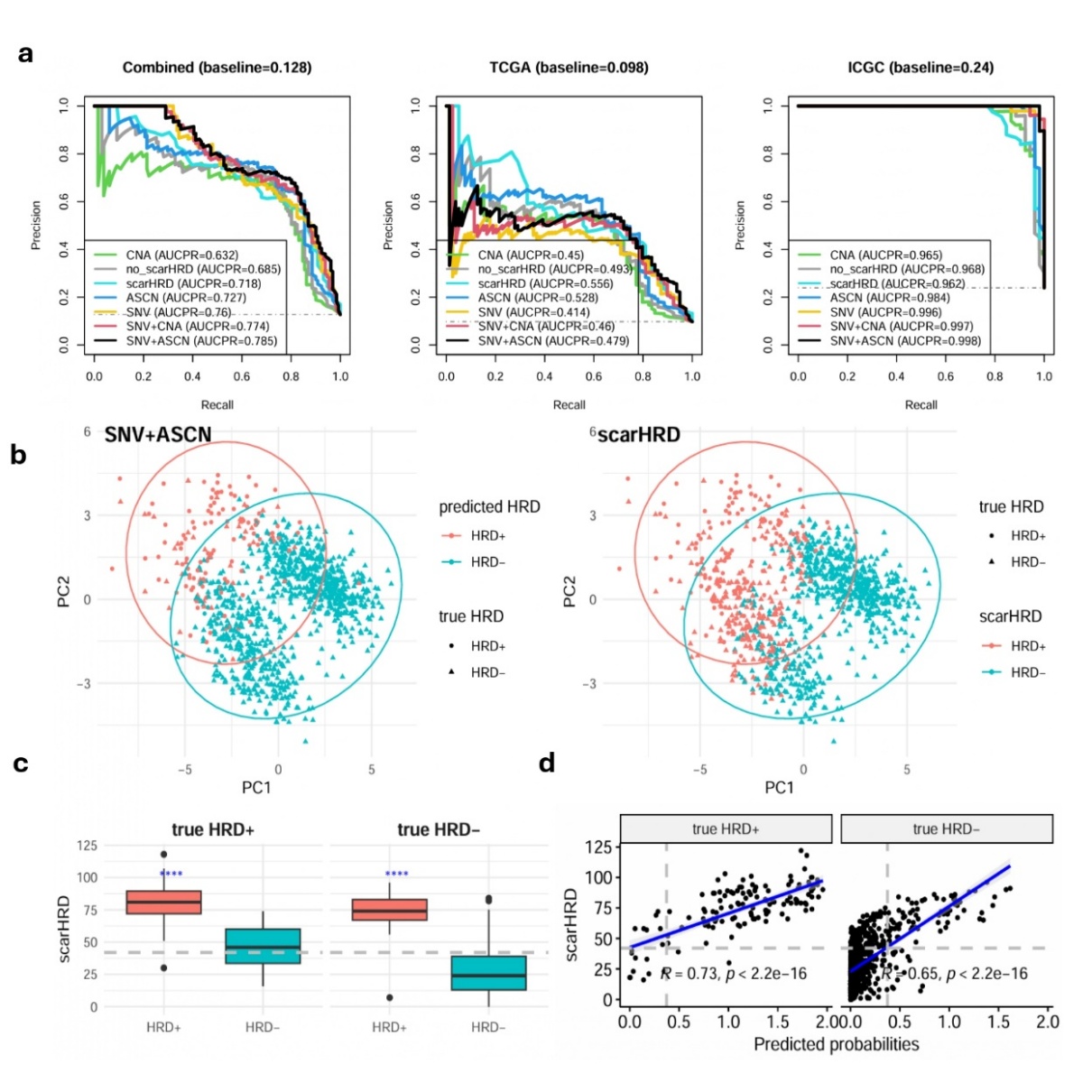


**Supplementary Figure 5**: Boxplots of predicted HRD probabilities computed using Leave-one-out cross-validation (LOOCV) among HRD+ and HRD– groups in the combined and two training cohorts separately (Wilcoxon rank-sum test, ****P < 0.0001; the dashed line represents the optimal threshold.).


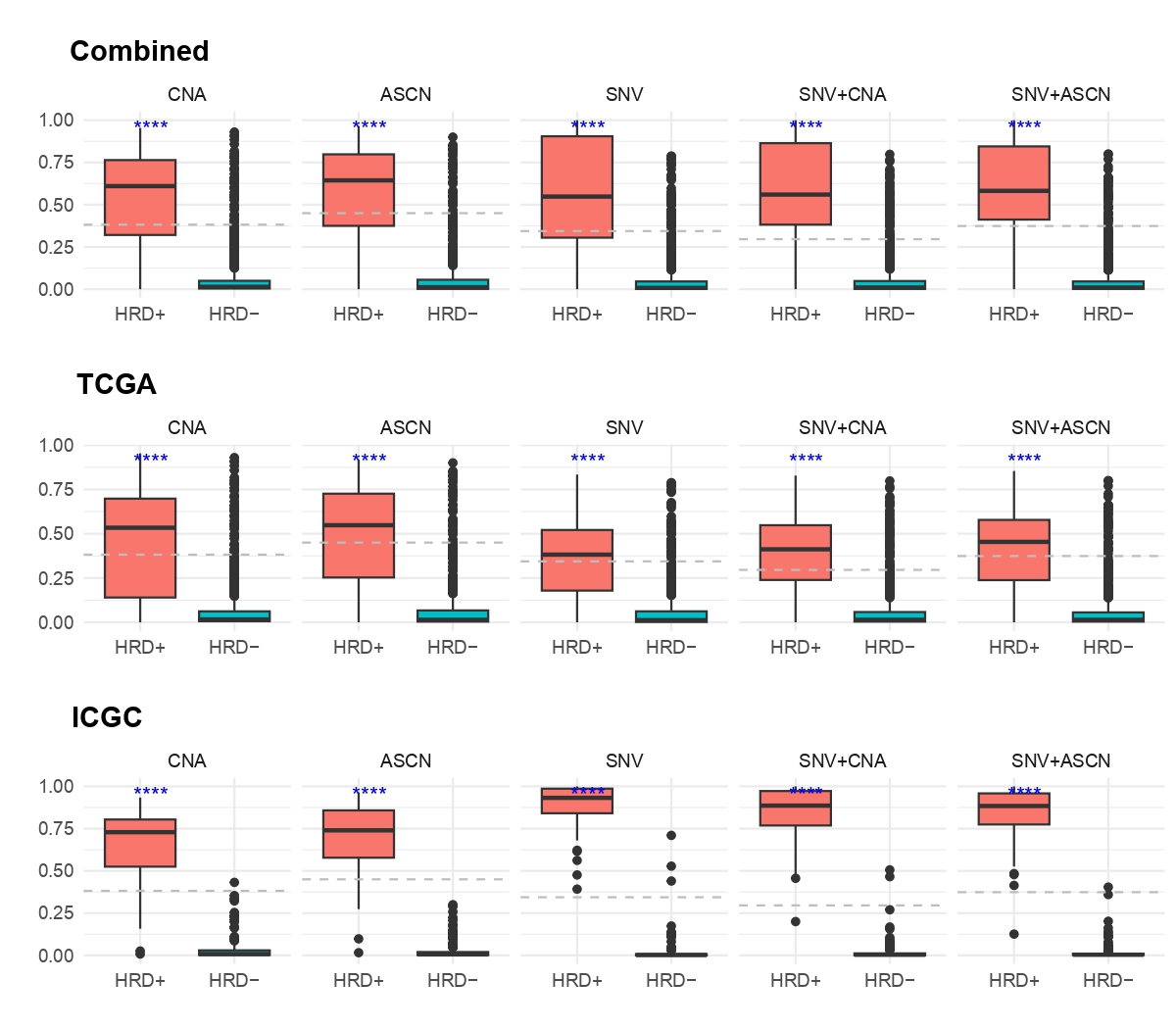


**Supplementary Figure 6**: Predicted probabilities for the training observations in the combined dataset in the five models, sorted from smallest to largest and coloured by HRD status. Unlabelled cases are represented in black, HRD– cases in light grey, and HRD+ cases in various colours. The dashed line represents the optimal threshold. (LOH: Loss of heterozygosity)


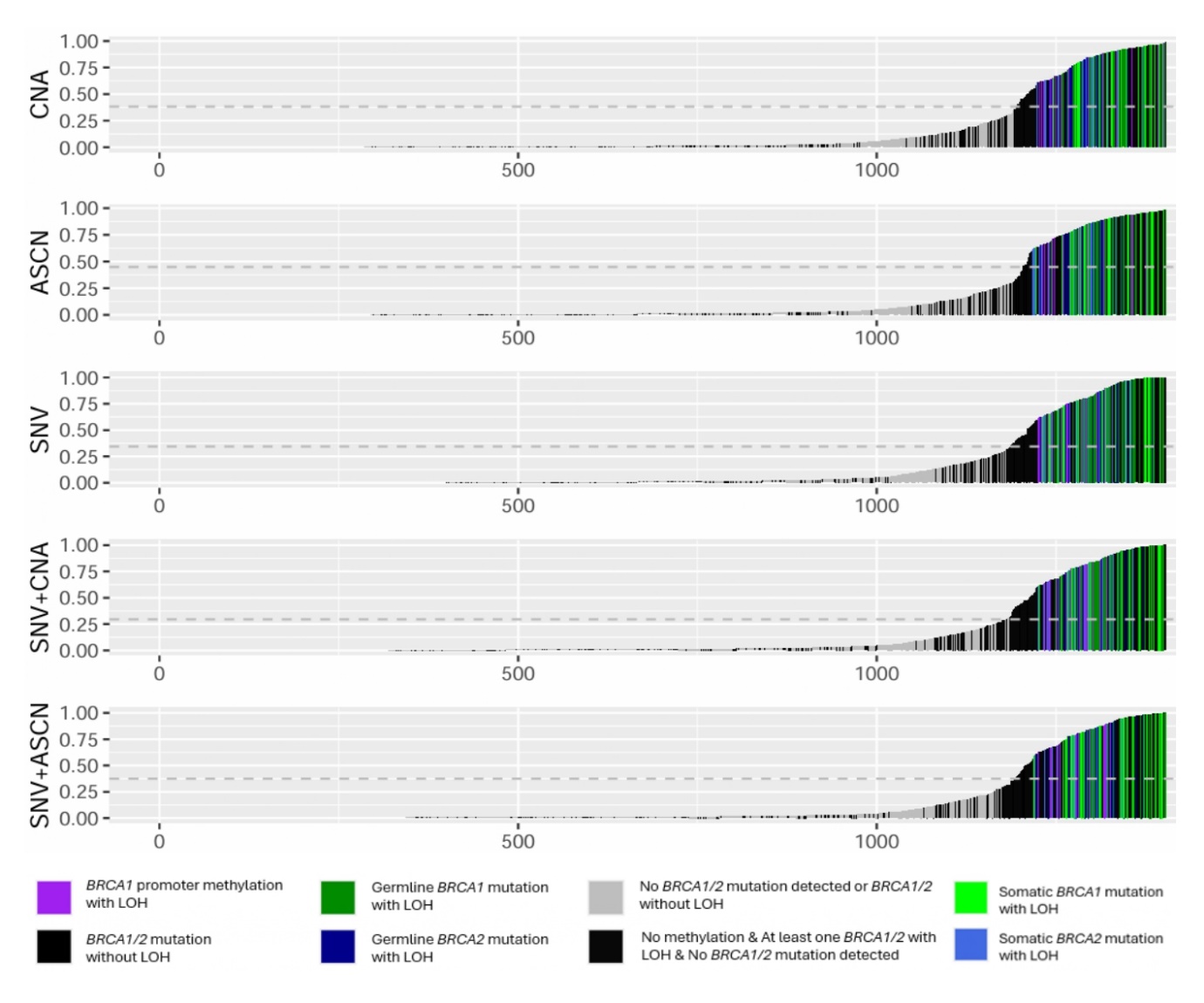


**Supplementary Figure 7:** Pearson’s product moment correlation coefficient between six features of HRDetect and selected features of three blocks in the GEL set, where the non-significant results are blank.


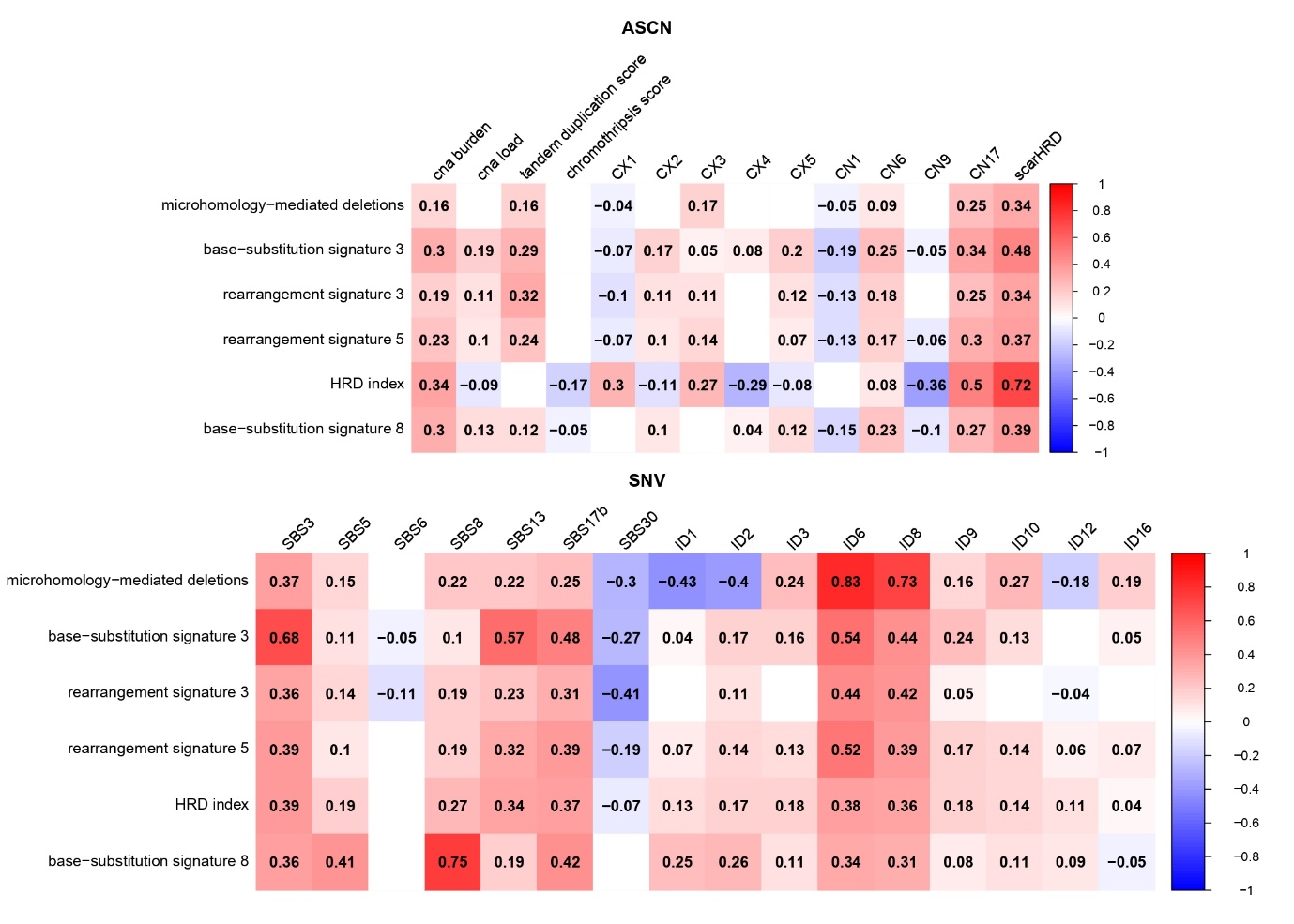


**Supplementary Figure 8:** Pearson’s product-moment correlation coefficient between the selected features among and within the three blocks in the combined dataset, where the non-significant results are blank. (a) shows the correlation between features in CNA and SNV features, (b) between ASCN and SNV features, (c) between CNA and ASCN features, (d) between ASCN features, (e) between SNV features, and (f) between CNA features.


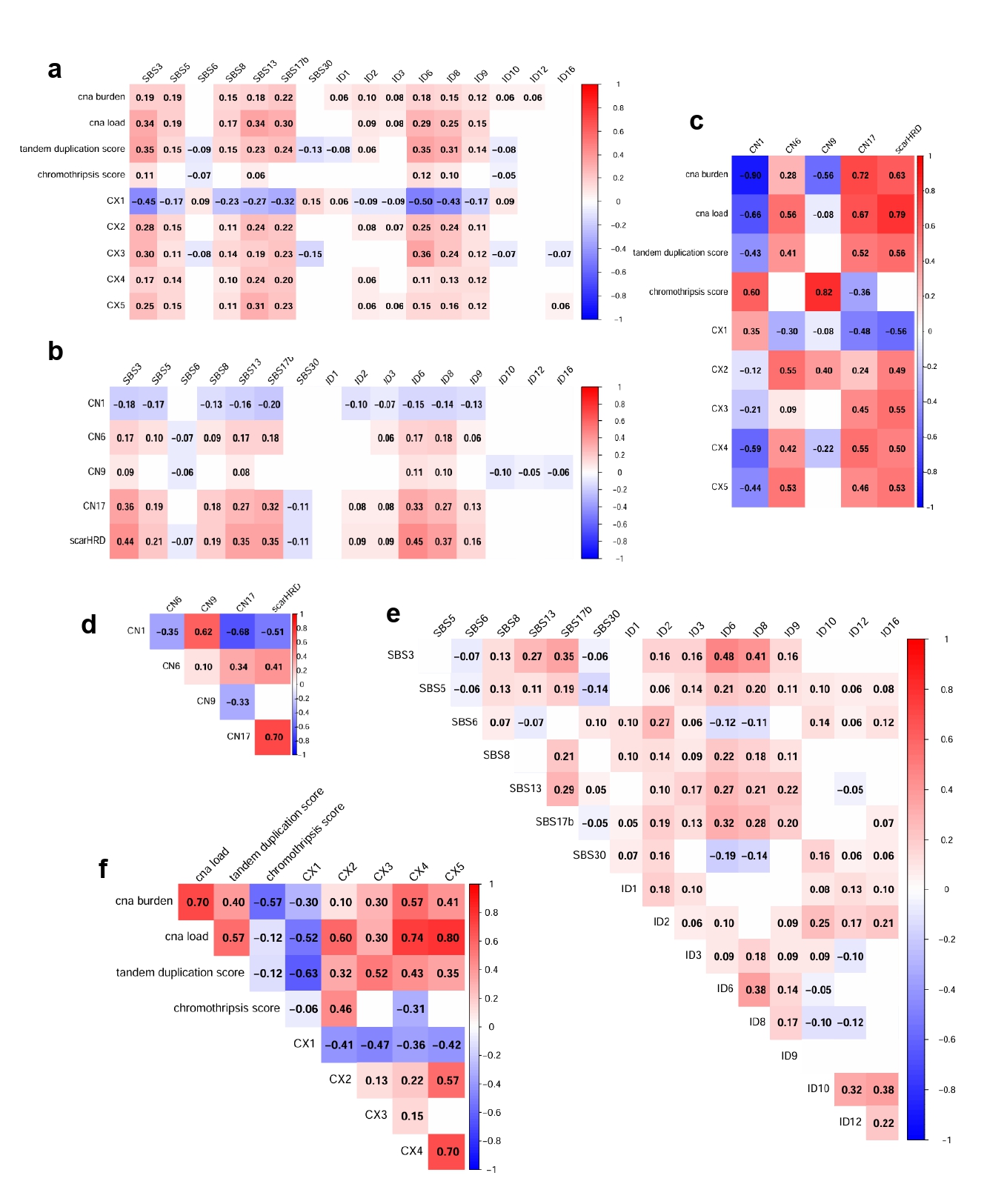


**Supplementary Figure 9:** (a) Boxplots of predicted HRD probabilities for five models among HRD+ and HRD– groups in TransNEO (Wilcoxon rank-sum test, ‘ns’=non-significant, **P < 0.01, ***P < 0.001; dashed line is the optimal threshold.). (b) Scatter plots between predicted HRD probabilities generated by five models and scarHRD scores on 25 breast cancer cell lines from the CCLE dataset and their sensitivity to different platinum-based chemotherapies as assessed through the Profiling relative inhibition simultaneously in mixtures (PRISM) metric. Pearson correlation coefficients and p-values are shown. The vertical dashed lines represent the optimal thresholds for the five models, as well as the scarHRD score threshold of 42.


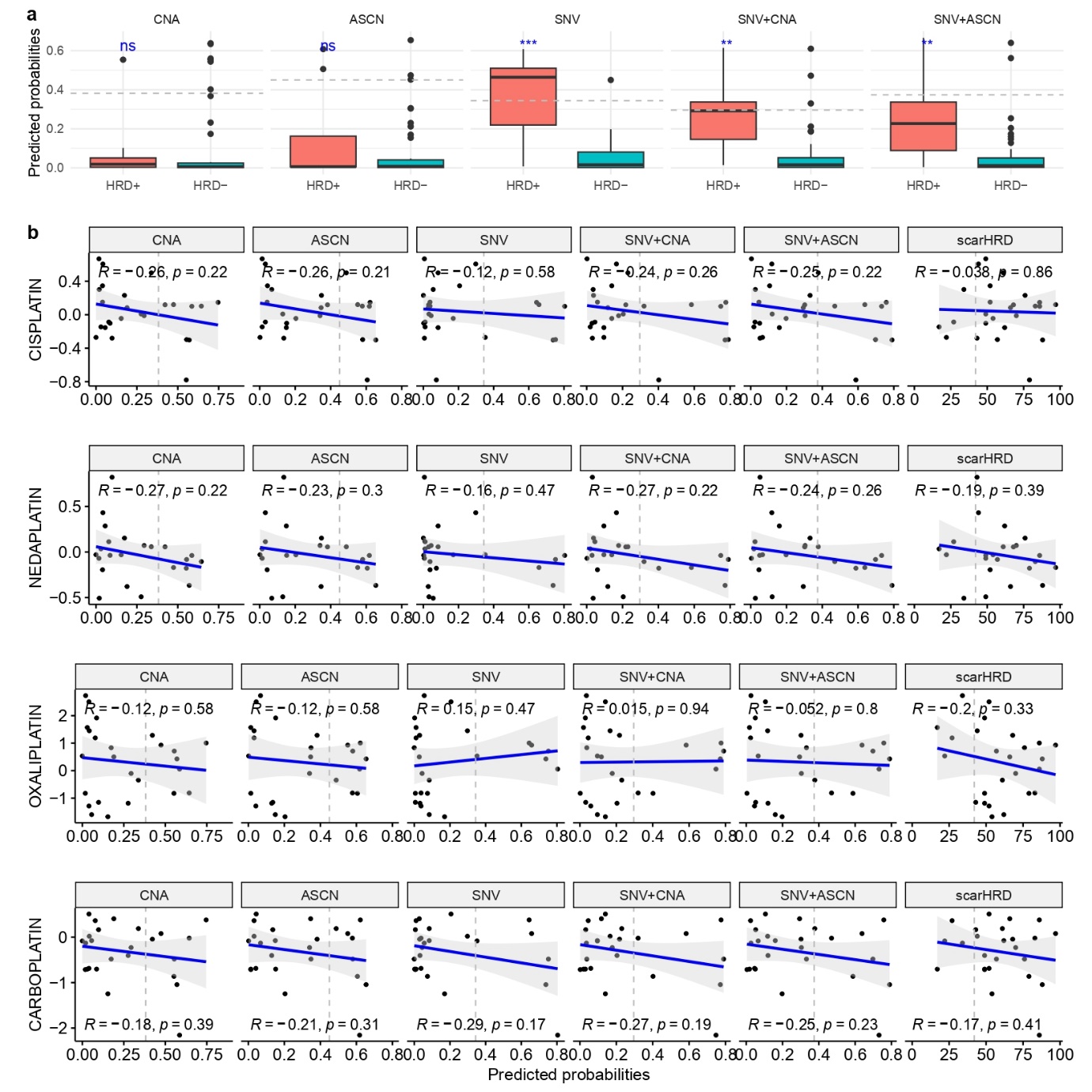


**Supplementary Figure 10**: Characteristics of tumours stratified based on predicted HRD-positive (HRD+) and HRD-negative (HRD–) status across five models in TCGA, ICGC, METABRIC, GEL, SCAN-B, TransNEO and MyBrCa (Models 1, 3 and 4). Note that the SCAN-B cohort only contains triple negative tumours. Subtypes in the Pam50 (Prediction analysis of microarray 50) classification system are abbreviated as Ba (basal-like), H (Her2-enriched), A (luminal A), B (luminal B), and N (normal-like). (IntClust: Integrative clustering; LN: Lymph node; PR: Progesterone receptor; Her2: Human epidermal growth factor receptor 2; ER: Estrogen receptor.)


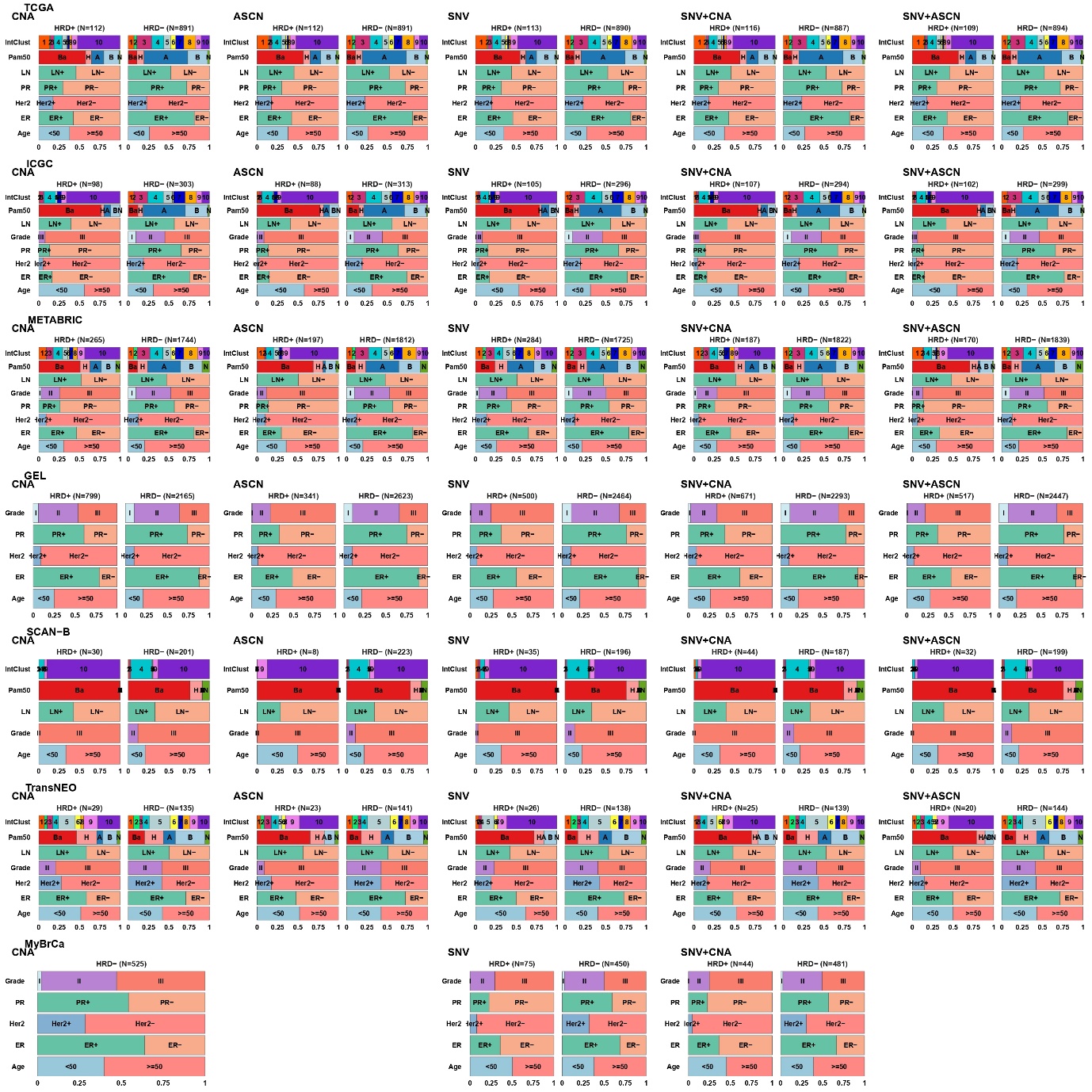


**Supplementary Figure 11**: Proportions of somatic mutations in tumours stratified based on the *BRCA1/2* status (true label) and the predicted HRD-positive (HRD+) and HRD-negative (HRD–) status across five models in the combined TCGA and ICGC cohorts.


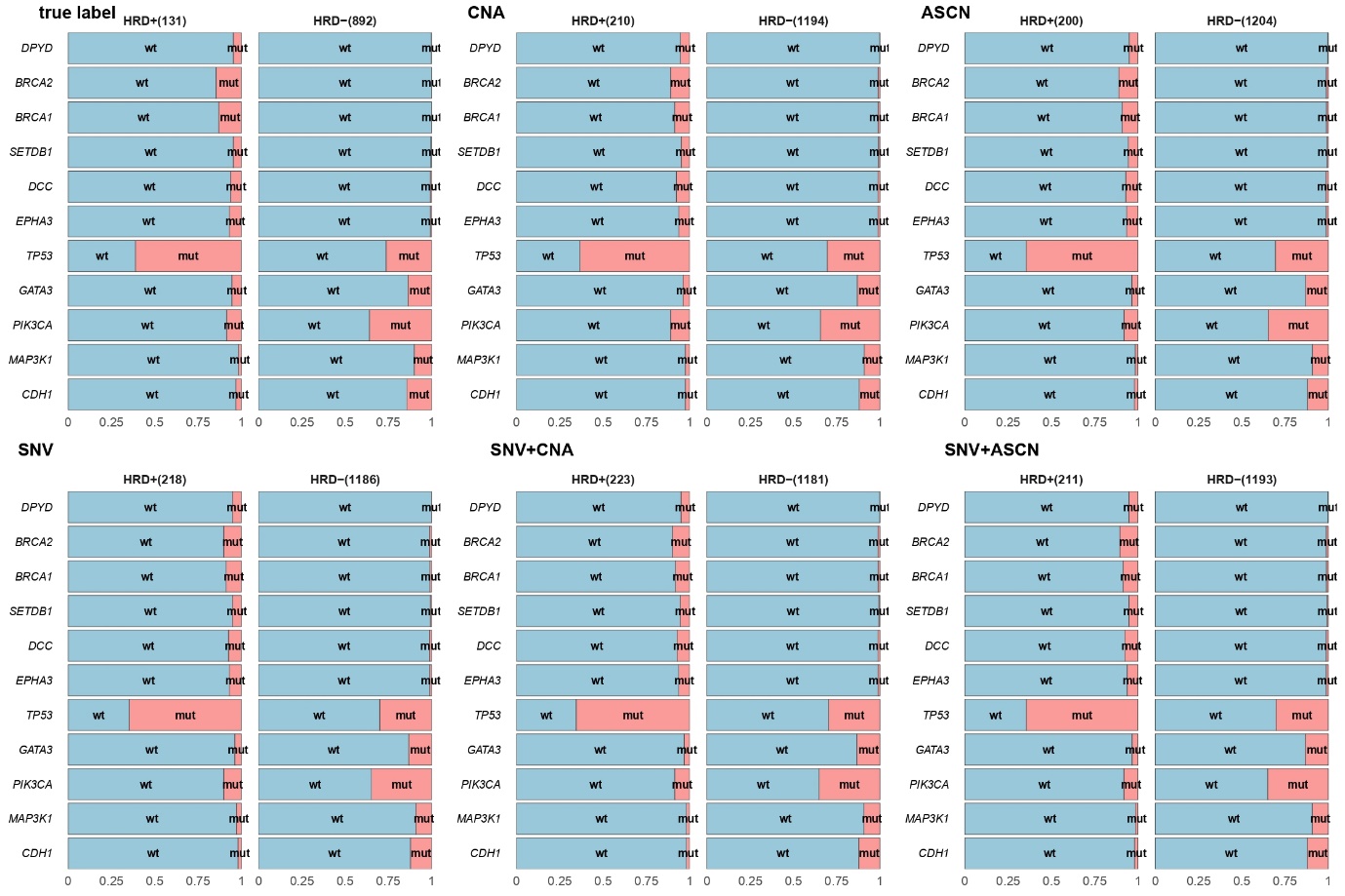

Supplement: Supplementary file 1 — Fig. S1. A summary of the five trained models categorised by the features they incorporate. Fig. S2. Boxplots and Wilcoxon rank‐sum test results (‘ns’ = non‐significant, *P < 0.05, **P < 0.01, ***P < 0.001, ****P < 0.0001) of all features from three blocks in combined datasets for HRD status. Fig. S3. Receiver Operating Characteristic (ROC) curves for Leave‐one‐out cross‐validation (LOOCV) predicted probabilities across five models based on the HRD status within the combined cohorts and each individual cohort. The first row displays results from our random forest‐based self‐training methods (same as Figure 3a), while the second and third rows show results from gradient boosting and logistic regression‐based self‐training methods, respectively. Fig. S4. (a) Precision‐Recall (PR) curves for Leave‐one‐out cross‐validation (LOOCV) predicted probabilities in seven models based on the HRD status within the combined two cohorts and each cohort. The no_scarHRD model incorporates relative copy number features and ASCN features while excluding the scarHRD score, whereas the scarHRD model consists solely of the scarHRD score. The dashed baseline is determined by the ratio of HRD positives (P) and negatives (N) as y = P/(P + N). (b) Principal component analysis (PCA) visualisation of the first two principal components (PC1 and PC2) based on samples from the combined cohorts, excluding those with unknown HRD status. Data points are distinguished by shape based on their true HRD status, and colour‐coded according to their predicted HRD status, derived from our fifth model and the scarHRD method, shown separately. Ellipses represent the two clusters formed according to the true HRD status. (c) Boxplots and Wilcoxon rank‐sum test results (****P < 0.0001) comparing scarHRD scores between the two HRD categories predicted by our fifth model among samples with true HRD+ and true HRD− in the combined cohorts, shown separately. The horizontal dashed line represents the scarHRD score thre [file MOL2-19-3613-s001.docx]
